# Grain and dietary fiber intake and bladder cancer risk: a pooled analysis of prospective cohort studies

**DOI:** 10.1093/ajcn/nqaa215

**Published:** 2020-08-10

**Authors:** Evan Y W Yu, Anke Wesselius, Siamak Mehrkanoon, Maree Brinkman, Piet van den Brandt, Emily White, Elisabete Weiderpass, Florence Le Calvez-Kelm, Marc Gunter, Inge Huybrechts, Fredrik Liedberg, Guri Skeie, Anne Tjonneland, Elio Riboli, Graham G Giles, Roger L Milne, Maurice P Zeegers

**Affiliations:** Department of Complex Genetics and Epidemiology, School of Nutrition and Translational Research in Metabolism, Maastricht University, Maastricht, Netherlands; Department of Complex Genetics and Epidemiology, School of Nutrition and Translational Research in Metabolism, Maastricht University, Maastricht, Netherlands; Department of Data Science and Knowledge Engineering, Maastricht University, Maastricht, Netherlands; Department of Complex Genetics and Epidemiology, School of Nutrition and Translational Research in Metabolism, Maastricht University, Maastricht, Netherlands; Department of Clinical Studies and Nutritional Epidemiology, Nutrition Biomed Research Institute, Melbourne, Victoria, Australia; Cancer Epidemiology Division, Cancer Council Victoria, Melbourne, Victoria, Australia; Department of Epidemiology, School for Oncology and Developmental Biology, Maastricht University Medical Centre, Maastricht, Netherlands; Department of Epidemiology, School for Public Health and Primary Care, Maastricht University Medical Centre, Maastricht, Netherlands; Fred Hutchinson Cancer Research Center, Seattle, WA, USA; International Agency for Research on Cancer/WHO, Lyon, France; International Agency for Research on Cancer/WHO, Lyon, France; International Agency for Research on Cancer/WHO, Lyon, France; International Agency for Research on Cancer/WHO, Lyon, France; Department of Urology, Skåne University Hospital, Malmö, Sweden; Institution of Translational Medicine, Lund University, Malmö, Sweden; Department of Community Medicine, UIT The Arctic University of Norway, Tromsø, Norway; Danish Cancer Society Research Center, Copenhagen, Denmark; Department of Public Health, University of Copenhagen, Copenhagen, Denmark; Department of Epidemiology and Biostatistics, School of Public Health, Imperial College London, London, United Kingdom; Cancer Epidemiology Division, Cancer Council Victoria, Melbourne, Victoria, Australia; Centre for Epidemiology and Biostatistics, Melbourne School of Population and Global Health, The University of Melbourne, Melbourne, Victoria, Australia; Precision Medicine, School of Clinical Sciences at Monash Health, Monash University, Clayton, Victoria, Australia; Cancer Epidemiology Division, Cancer Council Victoria, Melbourne, Victoria, Australia; Centre for Epidemiology and Biostatistics, Melbourne School of Population and Global Health, The University of Melbourne, Melbourne, Victoria, Australia; Precision Medicine, School of Clinical Sciences at Monash Health, Monash University, Clayton, Victoria, Australia; Department of Complex Genetics and Epidemiology, School of Nutrition and Translational Research in Metabolism, Maastricht University, Maastricht, Netherlands; CAPHRI School for Public Health and Primary Care, Maastricht University, Maastricht, Netherlands; School of Cancer Sciences, University of Birmingham, Birmingham, United Kingdom

**Keywords:** bladder cancer, grain, dietary fiber, dose-response analysis, cohort study

## Abstract

**Background:**

Higher intakes of whole grains and dietary fiber have been associated with lower risk of insulin resistance, hyperinsulinemia, and inflammation, which are known predisposing factors for cancer.

**Objectives:**

Because the evidence of association with bladder cancer (BC) is limited, we aimed to assess associations with BC risk for intakes of whole grains, refined grains, and dietary fiber.

**Methods:**

We pooled individual data from 574,726 participants in 13 cohort studies, 3214 of whom developed incident BC. HRs, with corresponding 95% CIs, were estimated using Cox regression models stratified on cohort. Dose–response relations were examined using fractional polynomial regression models.

**Results:**

We found that higher intake of total whole grain was associated with lower risk of BC (comparing highest with lowest intake tertile: HR: 0.87; 95% CI: 0.77, 0.98; HR per 1-SD increment: 0.95; 95% CI: 0.91, 0.99; *P* for trend: 0.023). No association was observed for intake of total refined grain. Intake of total dietary fiber was also inversely associated with BC risk (comparing highest with lowest intake tertile: HR: 0.86; 95% CI: 0.76, 0.98; HR per 1-SD increment: 0.91; 95% CI: 0.82, 0.98; *P* for trend: 0.021). In addition, dose–response analyses gave estimated HRs of 0.97 (95% CI: 0.95, 0.99) for intake of total whole grain and 0.96 (95% CI: 0.94, 0.98) for intake of total dietary fiber per 5-g daily increment. When considered jointly, highest intake of whole grains with the highest intake of dietary fiber showed 28% reduced risk (95% CI: 0.54, 0.93; *P* for trend: 0.031) of BC compared with the lowest intakes, suggesting potential synergism.

**Conclusions:**

Higher intakes of total whole grain and total dietary fiber are associated with reduced risk of BC individually and jointly. Further studies are needed to clarify the underlying mechanisms for these findings.

## Introduction

Bladder cancer (BC) is the 10th most common malignancy worldwide, with an estimated 550,000 new cases and 200,000 deaths annually (1, 2). Incidence rates of BC are highest in Europe and North America, with a strong predominance in males and the elderly (3–8). BC is reported to be the most expensive of all cancers in terms of lifetime treatment owing to its high rate of recurrence (9). Diet has been suspected to be important, in addition to smoking and occupational exposure, but only arsenic-contaminated food is considered to be an established dietary risk factor for BC (10–14). Because grain intake is an important component of numerous dietary guidelines globally, interest in the health effects of grain intake is increasing (15, 16).

Whole grains contain all components of the kernel, i.e., the bran, germ, and endosperm. Both the bran outer coating and the inner germ are major sources of dietary fiber, vitamins, minerals, phytonutrients, and numerous other nutrients which may be beneficial to health (17). However, during the refining process, the outer bran and inner germ are removed and only the endosperm is retained. This results in a substantial reduction in dietary fiber, vitamins, minerals, and other components. Although many vitamins and minerals are often added back to refined grains by subsequent processing, the fiber content remains greatly diminished (18, 19).

An accumulation of evidence shows that intake of dietary fiber is associated with lower risk of insulin resistance, hyperinsulinemia (20), and inflammation (21), which are known predisposing factors for cancer (22); however, evidence of association with BC risk is sparse, with only 2 case-control studies reporting insufficient evidence of an inverse association for intake of whole grains (23, 24). In contrast to the beneficial health associations of whole grains containing rich fiber, studies of refined grains mainly show no association with health (25–29), or harmful associations (30, 31), and there is no strong evidence of association with BC risk.

We therefore assessed associations with BC risk for intakes of whole grains and refined grains, using data from 13 prospective cohort studies pooled in the BLEND (BLadder cancer Epidemiology and Nutritional Determinants) international study. In addition, we also investigated the potential association of dietary fiber intake with BC risk by evaluating total and individual food sources (i.e., cereal, fruit, and vegetable fiber).

## Methods

### Study sample

Data were obtained from BLEND, an international nutritional consortium currently consisting of 19 case-control studies and 16 cohort studies. Thirteen cohort studies with a total of 574,726 participants, 3214 of whom developed incident BC, had sufficient information on grain intake to be eligible for inclusion in the present study (**Supplementary Figure 1**). These studies originated from 12 countries in 3 continents {i.e., Europe: EPIC [European Prospective Investigation into Cancer and Nutrition cohort study] (32) [Denmark (33), France (34), Germany (35), Greece (36), Italy (37), Spain (36), Sweden (38, 39), the Netherlands (40), the United Kingdom (41, 42), and Norway (43)] and NLCS (NetherLands Cohort Study) (44); North America: VITAL (VITamins And Lifestyle cohort study) in the United States (45); and Oceania: MCCS (Melbourne Collaborative Cohort Study) in Australia (46, 47)}. Person-years of follow-up for each participant were calculated from the date of study enrolment until the date of BC diagnosis or the date of last follow-up (e.g., date of death, lost to follow-up, or study exit), whichever came first. For the NLCS study, a nested case-cohort design was applied in order to increase the follow-up coverage and efficiency, in which the number of person-years at risk was estimated based on a subcohort that was randomly sampled (44). Each study was approved by their local ethical research committee (32, 44, 45, 47) (**Supplemental Table 1**). Informed consent was obtained from all individual participants included in each study.

### Data collection and coding

Details on the methodology of the BLEND consortium have been described elsewhere (48). In brief, all included studies used a self-administered or trained interviewer–administered FFQ that was validated on either food groups (45, 49–52) and/or energy intake (49, 52, 53). For each study, participants were asked to report on their usual intake during the year before study enrolment of individual types of whole grains [i.e., brown rice, wheat or oat, and basic products of other cereals (e.g., buckwheat, millet, sorghum, or spelt)] and of refined grains [i.e., white rice, pasta or noodles, leavened bread, unleavened bread, other bakery wares, savory cereal dishes (e.g., dumplings, couscous, risotto, pizza, pancake, or pie), and breakfast cereals]. These data were harmonized using the hierarchal Eurocode 2 food coding system developed by the European Union (54), with weekly, monthly, or yearly intake converted to grams per day. This resulted in an aggregated data set with unified dietary intakes across the different studies included. In order to extract dry weight (e.g., uncooked pasta or noodles, uncooked rice, uncooked wheat or oat) across all grains, the water content of grains was determined according to the composition database from the USDA and subtracted from the grain intake (55). Total intakes of dietary fiber and dietary fiber from cereal, fruit, and vegetables were calculated by multiplying the amount of each food consumed by the dietary fiber content per gram according to the USDA.

Each study ascertained incident BC, defined to include all urinary bladder neoplasms according to the International Classification of Diseases for Oncology, third edition (code C67), using population-based cancer registries, health insurance records, or medical records. BCs were classified as nonmuscle invasive bladder cancer (NMIBC) or muscle invasive bladder cancer (MIBC). For the present study, the primary outcome was defined as BC cases or non-BC cases, and the secondary outcome was defined as NMIBC, MIBC, or non-BC cases. NMIBC included noninvasive papillary carcinomas confined to the urothelium (stage Ta) and carcinomas that invaded the lamina propria of the bladder wall (stage T1). High-grade flat noninvasive carcinomas confined to the urothelium (carcinoma in situ) without other concomitant tumor stages [i.e., T1/Ta (classified to nonmuscle invasive prior) or muscle invasive] were also classified as NMIBC. MIBC included carcinomas that invaded into the detrusor muscle (stage T2), carcinomas that invaded into the peri-vesical tissue (stage T3), and carcinomas that invaded adjacent tissues and organs (most often the prostate or uterus, stage T4).

In addition to information on grain and other dietary intakes, the BLEND data set also included data on study characteristics (design, method of dietary assessment, geographical region), participant demographics (age, sex, and ethnicity), smoking status, and smoking pack-years (i.e., the number of cigarettes smoked per day multiplied by the years of smoking), which were measured at baseline.

### Statistical analyses

To assess the influence of intake of grains and fiber on BC risk, Cox regression analyses with a stratification approach to adjust for cross-cohort heterogeneity (56) were used to estimate the pooled HRs and 95% CIs. The proportional hazard assumption was examined for each analysis and no evidence of violation was found. In addition, the appropriateness of the use of the log-normal distribution was tested using a Wald test, and again no evidence of violation was found. Grain intake (i.e., total grain, total whole grain, total refined grain, brown rice, wheat or oat, basic products of other cereals, white rice, pasta or noodles, leavened bread, unleavened bread, bakery wares, savory cereals, and breakfast cereals) and dietary fiber intake (i.e., total dietary fiber from all food sources, cereal fiber, fruit fiber, and vegetable fiber) were divided into 3 groups defined by tertile based on the pooled data: low intake (tertile 1), medium intake (tertile 2), and high intake (tertile 3). Low intake was used as the reference group and associations were assessed applying 2 models: model 1 adjusted for age (y), sex (male/female), smoking, and total energy intake [kcal/d; continuous; using a residual model to remove extraneous variation (57)] and included cohort as a stratification variable (**Supplemental Tables 2–5** provide results) and model 2 in addition adjusted for ethnicity (Caucasian/non-Caucasian) and for potential dietary factors that affect the development of BC (10), including alcohol intake (mL/d; continuous), sugar intake (g/d; continuous), meat intake (g/d; continuous), vegetable intake (g/d; continuous), fruit intake (g/d; continuous), fat intake (g/d; continuous), and total fluid intake (mL/d; continuous). Smoking was defined as a dummy variable as follows: 0 (never smokers); 1 [current light smokers (i.e., smoking <20 pack-years)]; 2 [current heavy smokers (i.e., smoking >20 pack-years)]; 3 [current smokers (no information on pack-years)]; 4 [former light smokers (i.e., smokers who ceased smoking >1 y prior and smoked <20 pack-years)]; 5 [former heavy smokers (i.e., smokers who ceased smoking >1 y prior and smoked >20 pack-years)]; and 6 [former smokers (smokers who ceased smoking >1 y prior and no information on pack-years)]. The main interaction terms (between grain/dietary fiber and age, sex, and smoking; between total whole grain and total dietary fiber) were added to model 1 (*P*-interaction < 0.05 was considered statistically significant). Stratified analyses were performed by BC subtype (i.e., NMIBC and MIBC), sex, and smoking status. In addition, the HRs and 95% CIs of BC per 1-SD increase in grain and dietary fiber intakes were also estimated using the same models. Furthermore, a potential joint association of total whole grain and total dietary fiber intakes with BC risk was assessed using the lowest intakes of both total whole grain and total dietary fiber as the reference. To test for linearity or nonlinearity, we included both linear and quadratic terms (i.e., the absolute intake and intake squared) in the models, then a likelihood ratio test was used to assess the difference between the nonlinear and linear models (58). Because results showed no evidence of a nonlinear association, linear models were applied in the present study. A *P* for trend test was conducted by assigning medians to per 1 SD as a continuous variable in the models. The variables of BC status (i.e., cases or noncases), follow-up time, age, sex, smoking, ethnicity, and total energy intake were complete without missing values. Missing variables (e.g., alcohol intake, sugar intake, meat intake, vegetable intake, fruit intake, fat intake, and total fluid intake; missing proportions were all <5%) were imputed separately in each participating cohort by the multiple imputation method. Only participants with complete information on BC status, age, sex, smoking, ethnicity, and total energy intake were included when building the imputation models. Linear regression models were then fitted for those variables with missing data separately.

In our secondary analyses, potential dose–response relations of grain/dietary fiber with BC risk were assessed by using fractional polynomial regression from the ln of the HRs across categories of intake, in which the best-fitting second-order fractional polynomial regression model was defined as the model with the lowest deviance (59, 60). For this, we categorized each source of grain (e.g., total whole grain or total refined grain) and dietary fiber (e.g., total dietary fiber, cereal fiber, fruit fiber, and vegetable fiber) into 10 doses according to the range of intake of each grain or dietary fiber, by which the intervals of each intake were different. *P* values for trend were estimated by assigning medians to each category of intake as a continuous variable. A likelihood ratio test was used to assess the difference between the nonlinear (i.e., the absolute dose and dose squared) and linear (i.e., the absolute dose) models to test for linearity or nonlinearity (58). Model 2 was applied in dose–response analyses.

Sensitivity analyses were performed by *1*) removing cases diagnosed within the first 2 y after recruitment to each study and *2*) only including the complete data set, thereby excluding the participants with missing data on variables included in model 2. An extra sensitivity analysis for total refined grain was assessed by excluding pasta source in order to test whether the possible misclassification of pasta would influence the result. Furthermore, the role of smoking was tested by replacing the smoking dummy variable by both smoking status (never, former, and current) and smoking pack-years (continuous). In addition, a quintile-based analysis was performed in order to test whether the differently categorized intakes would affect the results. As a last step, the associations between intake of grains/dietary fiber and BC risk were assessed in each participating cohort separately and combined in a meta-analysis using a random-effect model; subsequently, we conducted a sensitivity analysis by excluding the study that mostly likely dominated the analysis for each dietary factor examined in the present study.

All statistical analyses were performed using STATA version 14 SE (Stata Corporation). A 2-tailed *P* value < 0.05 was considered statistically significant.

## Results

### Baseline characteristics

**Table 1** shows the baseline characteristics of the study sample. In total, 574,726 study participants contributed 6,335,667 person-years of follow-up over a median of 11 y, with 3214 incident BC cases (2416 males, 798 females) diagnosed. Of these, 2041 (63%) cases had available diagnosis records of NMIBC (39%) or MIBC (24%). The median age at baseline was 53 y. The majority (99.3%) of participants were Caucasian. No statistical interaction with age, sex, and smoking was found for total whole grain and total dietary fiber. Total refined grain intake showed a significant interaction with sex (*P*-interaction = 0.048).

**TABLE 1:** Characteristics of the study population (3214 cases and 571,512 noncases) according to tertiles of grain and dietary fiber intakes^1^

	Total whole grain					Total refined grain					Total dietary fiber				
	Low (tertile 1)	Medium (tertile 2)	High (tertile 3)	Mean ± SD, g/d	*P*-interaction	Low (tertile 1)	Medium (tertile 2)	High (tertile 3)	Mean ± SD, g/d	*P*-interaction	Low (tertile 1)	Medium (tertile 2)	High (tertile 3)	Mean ± SD, g/d	*P*-interaction
Overall population, *n* (%)	72,821 (33.5)	74,285 (34.1)	70,450 (32.4)	9.82 ± 12.69		191,576 (33.4)	191,575 (33.3)	191,575 (33.3)	156.37 ± 102.19		191,576 (33.4)	191,575 (33.3)	191,575 (33.3)	23.37 ± 12.38	
Noncases, *n* (%)	71,830 (33.3)	73,932 (34.3)	70,061 (32.4)	10.79 ± 14.19		190,572 (33.4)	190,337 (33.3)	190,603 (33.4)	156.39 ± 102.24		190,402 (33.3)	190,371 (33.3)	190,739 (33.4)	23.38 ± 12.40	
Cases, *n* (%)	991 (57.2)	353 (20.4)	389 (22.4)	9.81 ± 12.68		1004 (31.3)	1238 (38.5)	972 (30.2)	153.34 ± 93.32		1174 (36.5)	1204 (37.5)	836 (26.0)	21.04 ± 9.07	
TNM stage															
MIBC, *n* (%)	360 (63.7)	92 (16.3)	113 (20.0)	5.73 ± 11.50		267 (34.5)	356 (46.1)	150 (19.4)	135.49 ± 75.98		273 (35.3)	323 (41.8)	177 (22.9)	20.68 ± 8.56	
NMIBC, *n* (%)	424 (59.5)	133 (18.7)	156 (21.8)	6.78 ± 13.65		422 (33.3)	491 (38.7)	355 (28.0)	149.82 ± 97.02		472 (37.2)	467 (36.8)	329 (26.0)	20.84 ± 9.13	
Person-years	1,016,724	870,317	855,859	9.82 ± 12.69		1,929,466	2,262,417	2,143,784	156.38 ± 102.23		2,001,790	2,187,860	2,146,058	23.37 ± 12.38	
Sex					0.906					0.048					0.336
Male, *n* (%)	22,476 (36.1)	18,167 (29.2)	21,659 (34.7)	10.98 ± 14.52		57,331 (30.4)	51,474 (27.3)	80,057 (42.4)	177.41 ± 118.64		67,336 (35.7)	59,435 (31.5)	62,091 (32.8)	22.02 ± 10.68	
Female, *n* (%)	50,345 (32.4)	56,118 (36.2)	48,791 (31.4)	9.40 ± 11.93		134,245 (34.8)	140,101 (36.3)	111,518 (28.9)	146.08 ± 91.34		124,240 (32.2)	132,140 (34.3)	129,484 (33.6)	24.03 ± 13.09	
Age,^2^ y	52.98	49.75	48.51	9.82 ± 12.69	0.724	54.96	52.07	50.90	156.37 ± 102.19	0.406	54.93	51.86	51.14	23.37 ± 12.38	0.431
≤55, *n* (%)	40,336 (28.4)	50,475 (35.6)	50,994 (36.0)	10.22 ± 12.66		97,204 (28.6)	117,384 (34.5)	125,269 (36.9)	166.82 ± 104.19		97,474 (28.7)	117,242 (34.5)	125,141 (36.8)	24.52 ± 12.49	
55–60, *n* (%)	12,111 (37.8)	10,592 (33.1)	9300 (29.1)	9.04 ± 11.61		33,855 (34.5)	31,659 (32.3)	32,527 (33.2)	154.42 ± 102.14		33,998 (34.7)	32,299 (32.9)	31,744 (32.4)	23.03 ± 12.43	
60–65, *n* (%)	10,241 (41.8)	7687 (31.4)	6557 (26.8)	9.13 ± 12.47		28,094 (35.4)	26,446 (33.3)	24,846 (31.3)	149.48 ± 96.97		29,037 (36.6)	26,494 (33.4)	23,855 (30.0)	22.34 ± 11.85	
65–70, *n* (%)	6919 (52.1)	3653 (27.5)	2709 (20.4)	8.92 ± 13.95		18,125 (50.6)	11,241 (31.4)	6464 (18.0)	116.61 ± 83.20		17,708 (49.4)	10,284 (28.7)	7838 (21.9)	19.74 ± 11.78	
70–75, *n* (%)	2311 (54.7)	1305 (30.9)	610 (14.4)	8.08 ± 12.60		12,691 (68.5)	3917 (22.2)	1909 (10.3)	88.89 ± 68.98		12,691 (68.5)	3917 (22.2)	1909 (10.3)	15.85 ± 8.61	
>75, *n* (%)	903 (51.4)	573 (32.6)	280 (16.0)	8.15 ± 13.10		1607 (51.9)	928 (30.0)	560 (18.1)	111.71 ± 76.90		1331 (43.0)	897 (29.0)	867 (28.0)	21.23 ± 11.68	
Smoking status															
Never, *n* (%)	38,321 (32.0)	42,404 (35.4)	39,024 (32.6)	9.91 ± 12.91		97,586 (34.1)	98,375 (34.4)	90,409 (31.6)	152.51 ± 98.36		89,338 (31.2)	94,872 (33.1)	102,160 (35.7)	24.37 ± 13.21	
Former, *n* (%)	21,503 (36.3)	19,219 (32.4)	18,553 (31.3)	9.43 ± 12.97		62,296 (36.8)	52,397 (31.0)	54,517 (32.2)	151.42 ± 103.60		62,807 (37.1)	54,623 (32.3)	51,780 (30.6)	22.25 ± 11.83	
Current, *n* (%)	12,997 (33.7)	12,662 (32.9)	12,873 (33.4)	9.36 ± 11.52		31,694 (26.6)	40,803 (34.3)	46,649 (39.2)	172.70 ± 107.43		39,431 (33.1)	42,080 (35.3)	37,635 (31.6)	22.56 ± 10.79	
Smoking pack-years^3^	22.99	19.22	19.72	9.65 ± 12.30		24.50	22.66	23.08	150.29 ± 102.03		24.84	22.72	22.36	23.03 ± 12.67	
Smoking^4^					0.878					0.984					0.970
0, *n* (%)	38,321 (32.0)	42,404 (35.4)	39,024 (32.6)	9.91 ± 12.91		97,586 (34.1)	98,375 (34.4)	90,409 (31.6)	152.51 ± 98.36		89,338 (31.2)	94,872 (33.1)	102,160 (35.7)	24.37 ± 13.21	
1, *n* (%)	5526 (29.8)	6513 (35.2)	6489 (35.0)	9.55 ± 11.85		16,366 (28.9)	19,438 (34.3)	20,834 (36.8)	166.01 ± 104.24		18,745 (33.1)	20,112 (35.5)	17,781 (31.4)	22.49 ± 10.71	
2, *n* (%)	5930 (39.7)	4418 (29.6)	4594 (30.8)	8.95 ± 11.10		12,788 (24.8)	18,134 (35.1)	20,703 (40.1)	176.88 ± 109.31		17,486 (33.9)	18,410 (35.7)	15,729 (30.4)	22.31 ± 10.46	
3, *n* (%)	3555 (66.6)	693 (13.0)	1087 (20.4)	11.99 ± 16.22		22,466 (81.6)	4201 (15.3)	869 (3.2)	67.58 ± 46.92		19,501 (70.8)	5459 (19.8)	2576 (9.4)	14.31 ± 8.10	
4, *n* (%)	2505 (72.0)	368 (10.6)	606 (17.4)	12.53 ± 16.88		8996 (74.8)	2450 (20.4)	582 (4.8)	76.84 ± 52.01		8111 (67.4)	2612 (21.7)	1305 (10.9)	14.93 ± 8.31	
5, *n* (%)	1541 (30.4)	1731 (34.2)	1790 (35.4)	9.82 ± 11.33		2540 (23.3)	3231 (29.7)	5112 (47.0)	187.69 ± 112.19		3200 (29.4)	3558 (32.7)	4125 (37.9)	24.15 ± 12.48	
6, *n* (%)	15,443 (30.6)	18,158 (36.0)	16,860 (33.4)	9.73 ± 12.58		30,834 (23.8)	45,746 (35.3)	53,066 (40.9)	176.15 ± 103.30		35,195 (27.1)	46,552 (35.9)	47,899 (37.0)	24.61 ± 11.77	
Ethnicity															
Non-Caucasian,*n* (%)						3772 (94.0)	228 (5.7)	15 (0.3)	49.05 (31.28)		3302 (82.2)	514 (12.8)	199 (5.0)	11.88 (7.26)	
Caucasian,^5^*n* (%)	72,821 (33.5)	74,285 (34.1)	70,450 (32.4)	9.82 ± 12.69		187,804 (32.9)	191,347 (33.5)	191,560 (33.6)	157.13 ± 102.12		188,274 (33.0)	191,061 (33.5)	191,376 (33.5)	23.45 ± 12.37	
Energy intake, kcal/d	2055.90	2074.74	2251.09	9.82 ± 12.69		1731.56	2002.27	2455.98	156.37 ± 102.19		1726.74	2016.21	2446.86	23.37 ± 12.38	
Alcohol intake, mL/d	9.17	9.79	11.09	9.69 ± 12.43		7.20	9.57	12.44	157.61 ± 103.54		8.17	9.77	11.26	23.61 ± 12.39	
Fat intake, g/d	24.21	22.77	24.55	9.80 ± 12.69		16.88	25.30	31.74	156.57 ± 101.98		18.55	25.78	29.59	23.44 ± 12.52	
Fruit intake, g/d	154.49	147.67	160.53	9.83 ± 12.87		101.98	132.83	134.84	156.71 ± 101.48		72.61	118.01	179.02	23.37 ± 12.39	
Meat intake, g/d	85.93	82.24	88.92	9.80 ± 12.63		54.48	75.45	91.59	160.86 ± 101.79		54.50	73.94	93.09	23.75 ± 12.38	
Sugar intake, g/d	22.10	23.97	23.52	9.96 ± 13.06		20.73	23.32	27.37	155.95 ± 102.80		18.69	20.77	31.96	22.99 ± 11.97	
Vegetable intake, g/d	222.97	239.05	263.39	9.82 ± 12.69		181.77	212.32	204.19	156.68 ± 102.06		130.47	189.46	278.35	23.38 ± 12.38	
Total fluid intake, mL/d	1189.35	1264.41	1153.81	9.82 ± 12.69		1368.74	1337.53	1444.93	156.64 ± 102.07		1420.23	1397.25	1333.72	23.37 ± 12.38	

1 The intervals of tertiles were defined as follows: total whole grain: 0 ≤ tertile 1 ≤ 3 g/d, 3 < tertile 2 ≤ 8 g/d, tertile 3 > 8 g/d; total refined grain: 0 ≤ tertile 1 ≤ 102 g/d, 102 < tertile 2 ≤ 181 g/d, tertile 3 > 181 g/d; total dietary fiber: 0 ≤ tertile 1 ≤ 17 g/d, 17 < tertile 2 ≤ 25 g/d, tertile 3 > 25 g/d. *P*-interaction < 0.05 was considered statistically significant. MIBC, muscle invasive bladder cancer; NMIBC, nonmuscle invasive bladder cancer; TNM, tumor, nodes, and metastasis classification.

2 Age at the time of recruitment.

3 Pack-years was defined as the number of cigarettes smoked per day multiplied by the years of smoking.

4 Smoking was defined as a dummy variable: 0 (never smokers); 1 [current light smokers (i.e., smoking <20 pack-years)]; 2 [current heavy smokers (i.e., smoking >20 pack-years)]; 3 [current smokers (no information on pack-years)]; 4 [former light smokers (i.e., smokers who ceased smoking >1 y prior and smoked <20 pack-years)]; 5 [former heavy smokers (i.e., smokers who ceased smoking >1 y prior and smoked >20 pack-years)]; or 6 [former smokers (smokers who ceased smoking >1 y prior and no information on pack-years)].

5 All of the included participants for whole grain intake were Caucasian.

### Associations of grain and dietary fiber intakes with BC risk

#### Total grain intake and BC risk

For the different categories of intake of “total grains,” no evidence of association for tertile of intake was observed overall, by cancer subtype, by sex, or by smoking status (**Table 2**). However, the HR per 1-SD increment showed a decreased risk (model 2: 0.91; 95% CI: 0.85, 0.98; *P* for trend = 0.011) of BC among males.

**TABLE 2:** Risk of bladder cancer according to intakes of total grains, total whole grains, and total refined grains^1^

Grain source, subgroup, intake tertiles		Model 2^2^		
	Cases/participants, *n*	HR (95% CI)	HR per 1-SD increase (95% CI)	*P*-trend
Total grains, g/d				
Overall				
Tertile 1	1005/191,576	Ref.	0.97 (0.92, 1.02)	0.240
Tertile 2	1227/191,575	0.93 (0.84, 1.02)		
Tertile 3	982/191,575	0.94 (0.83, 1.05)		
MIBC				
Tertile 1	263/190,834	Ref.	0.90 (0.78, 1.02)	0.119
Tertile 2	350/190,698	0.89 (0.74, 1.08)		
Tertile 3	160/190,753	0.79 (0.61, 1.02)		
NMIBC				
Tertile 1	425/190,996	Ref.	0.99 (0.91, 1.07)	0.750
Tertile 2	481/190,829	0.96 (0.82, 1.13)		
Tertile 3	352/190,955	0.93 (0.77, 1.14)		
Male				
Tertile 1	793/62,954	Ref.	0.91 (0.85, 0.98)	0.011
Tertile 2	1049/62,954	0.89 (0.80, 1.00)		
Tertile 3	574/62,954	0.88 (0.77, 1.02)		
Female				
Tertile 1	287/128,622	Ref.	1.07 (0.97, 1.19)	0.174
Tertile 2	291/128,621	0.97 (0.81, 1.17)		
Tertile 3	220/128,621	1.10 (0.88, 1.37)		
Never smoker				
Tertile 1	212/95,457	Ref.	1.00 (0.90, 1.13)	0.801
Tertile 2	244/95,457	1.02 (0.79, 1.32)		
Tertile 3	201/95,456	0.94 (0.75, 1.17)		
Current smoker				
Tertile 1	403/39,716	Ref.	0.97 (0.89, 1.06)	0.488
Tertile 2	456/39,715	1.04 (0.90, 1.20)		
Tertile 3	339/39,715	0.99 (0.82, 1.19)		
Former smoker				
Tertile 1	416/56,404	Ref.	0.90 (0.82, 1.01)	0.517
Tertile 2	543/56,403	0.85 (0.72, 1.00)		
Tertile 3	400/56,403	0.85 (0.69, 1.02)		
Total whole grains, g/d				
Overall				
Tertile 1	991/72,821	Ref.	0.95 (0.91, 0.99)	0.023
Tertile 2	353/74,285	1.01 (0.89, 1.15)		
Tertile 3	389/70,450	0.87 (0.77, 0.98)		
MIBC				
Tertile 1	360/72,190	Ref.	0.92 (0.85, 1.00)	0.038
Tertile 2	92/74,024	1.21 (0.95, 1.53)		
Tertile 3	113/70,174	0.86 (0.70, 1.07)		
NMIBC				
Tertile 1	424/72,254	Ref.	0.96 (0.90, 1.03)	0.281
Tertile 2	133/72,065	1.07 (0.87, 1.32)		
Tertile 3	156/70,217	0.85 (0.70, 1.03)		
Male				
Tertile 1	787/22,476	Ref.	0.93 (0.83, 1.02)	0.059
Tertile 2	259/19,149	0.98 (0.84, 1.14)		
Tertile 3	295/20,677	0.85 (0.74, 0.98)		
Female				
Tertile 1	204/51,754	Ref.	0.93 (0.85, 1.01)	0.053
Tertile 2	104/51,830	0.98 (0.83, 1.15)		
Tertile 3	84/51,670	0.83 (0.71, 0.96)		
Never smoker				
Tertile 1	188/39,917	Ref.	0.96 (0.87, 1.06)	0.434
Tertile 2	93/40,808	1.04 (0.86, 1.45)		
Tertile 3	77/39,024	0.83 (0.63, 1.10)		
Current smoker				
Tertile 1	362/12,997	Ref.	0.96 (0.90, 1.02)	0.167
Tertile 2	117/12,699	1.00 (0.80, 1.25)		
Tertile 3	151/12,836	0.87 (0.71, 1.08)		
Former smoker				
Tertile 1	425/19,760	Ref.	0.94 (0.87, 1.02)	0.125
Tertile 2	152/19,970	0.98 (0.80, 1.19)		
Tertile 3	168/19,545	0.90 (0.75, 1.09)		
Total refined grains, g/d				
Overall				
Tertile 1	1004/191,576	Ref.	0.97 (0.92, 1.02)	0.242
Tertile 2	1238/191,575	0.93 (0.85, 1.03)		
Tertile 3	972/191,575	0.95 (0.84, 1.07)		
MIBC				
Tertile 1	267/190,839	Ref.	0.93 (0.81, 1.07)	0.327
Tertile 2	356/190,693	0.90 (0.74, 1.08)		
Tertile 3	150/190,753	0.80 (0.61, 1.04)		
NMIBC				
Tertile 1	422/190,994	Ref.	0.99 (0.91, 1.08)	0.906
Tertile 2	491/190,828	1.00 (0.85, 1.18)		
Tertile 3	355/190,958	0.98 (0.80, 1.20)		
Male				
Tertile 1	808/62,954	Ref.	0.92 (0.86, 1.00)	0.040
Tertile 2	1048/62,954	0.89 (0.80, 1.00)		
Tertile 3	560/62,954	0.87 (0.75, 1.01)		
Female				
Tertile 1	283/128,623	Ref.	1.08 (0.98, 1.20)	0.135
Tertile 2	295/128,620	1.00 (0.83, 1.20)		
Tertile 3	220/128,621	1.11 (0.89, 1.40)		
Never smoker				
Tertile 1	217/95,457	Ref.	0.99 (0.91, 1.10)	0.720
Tertile 2	232/95,457	0.92 (0.70, 1.08)		
Tertile 3	208/95,456	0.99 (0.81, 1.24)		
Current smoker				
Tertile 1	404/39,716	Ref.	0.98 (0.90, 1.07)	0.679
Tertile 2	464/39,715	1.07 (0.92, 1.24)		
Tertile 3	330/39,715	1.00 (0.83, 1.21)		
Former smoker				
Tertile 1	414/56,404	Ref.	0.91 (0.84, 1.00)	0.054
Tertile 2	552/56,403	0.89 (0.73, 1.07)		
Tertile 3	393/56,403	0.88 (0.75, 1.02)		

1 The intervals of tertiles were defined as follows: total grains: *1*) overall, 0 ≤ tertile 1 ≤ 105 g/d, 105 < tertile 2 ≤ 186 g/d, tertile 3 > 186 g/d; *2*) MIBC, 0 ≤ tertile 1 ≤ 105 g/d, 105 < tertile 2 ≤ 186 g/d, tertile 3 > 186 g/d; *3*) NMIBC, 0 ≤ tertile 1 ≤ 105 g/d, 105 < tertile 2 ≤ 186 g/d, tertile 3 > 186 g/d; *4*) male, 0 ≤ tertile 1 ≤ 113 g/d, 113 < tertile 2 ≤ 215 g/d, tertile 3 > 215 g/d; *5*) female, 0 ≤ tertile 1 ≤ 102 g/d, 102 < tertile 2 ≤ 173 g/d, tertile 3 > 173 g/d; *6*) never smoker, 0 ≤ tertile 1 ≤ 104 g/d, 104 < tertile 2 ≤ 181 g/d, tertile 3 > 181 g/d; *7*) current smoker, 0 ≤ tertile 1 ≤ 121 g/d, 121 < tertile 2 ≤ 204 g/d, tertile 3 > 204 g/d; *8*) former smoker, 0 ≤ tertile 1 ≤ 96 g/d, 96 < tertile 2 ≤ 182 g/d, tertile 3 > 182 g/d; total whole grains: *1*) overall, 0 ≤ tertile 1 ≤ 3 g/d, 3 < tertile 2 ≤ 8 g/d, tertile 3 > 8 g/d; *2*) MIBC, 0 ≤ tertile 1 ≤ 3 g/d, 3 < tertile 2 ≤ 8 g/d, tertile 3 > 8 g/d; *3*) NMIBC, 0 ≤ tertile 1 ≤ 3 g/d, 3 < tertile 2 ≤ 8 g/d, tertile 3 > 8 g/d; *4*) male, 0 ≤ tertile 1 ≤ 3 g/d, 3 < tertile 2 ≤ 9 g/d, tertile 3 > 9 g/d; *5*) female, 0 ≤ tertile 1 ≤ 3 g/d, 3 < tertile 2 ≤ 8 g/d, tertile 3 > 8 g/d; *6*) never smoker, 0 ≤ tertile 1 ≤ 3 g/d, 3 < tertile 2 ≤ 8 g/d, tertile 3 > 8 g/d; *7*) current smoker, 0 ≤ tertile 1 ≤ 3 g/d, 3 < tertile 2 ≤ 8 g/d, tertile 3 > 8 g/d; *8*) former smoker, 0 ≤ tertile 1 ≤ 3 g/d, 3 < tertile 2 ≤ 8 g/d, tertile 3 > 8 g/d; total refined grains: *1*) overall, 0 ≤ tertile 1 ≤ 102 g/d, 102 < tertile 2 ≤ 181 g/d, tertile 3 > 181 g/d; *2*) MIBC, 0 ≤ tertile 1 ≤ 102 g/d, 102 < tertile 2 ≤ 181 g/d, tertile 3 > 181 g/d; *3*) NMIBC, 0 ≤ tertile 1 ≤ 102 g/d, 102 < tertile 2 ≤ 181 g/d, tertile 3 > 181 g/d; *4*) male, 0 ≤ tertile 1 ≤ 111 g/d, 111 < tertile 2 ≤ 211 g/d, tertile 3 > 211 g/d; *5*) female, 0 ≤ tertile 1 ≤ 99 g/d, 99 < tertile 2 ≤ 169 g/d, tertile 3 > 169 g/d; *6*) never smoker, 0 ≤ tertile 1 ≤ 100 g/d, 100 < tertile 2 ≤ 176 g/d, tertile 3 > 176 g/d; *7*) current smoker, 0 ≤ tertile 1 ≤ 119 g/d, 119 < tertile 2 ≤ 201 g/d, tertile 3 > 201 g/d; *8*) former smoker, 0 ≤ tertile 1 ≤ 93 g/d, 93 < tertile 2 ≤ 178 g/d, tertile 3 > 178 g/d. Reference group was lowest intake (tertile 1). *P*-trend < 0.05 was considered statistically significant. MIBC, muscle invasive bladder cancer; NMIBC, nonmuscle invasive bladder cancer.

2 Model 2 of Cox regression: adjusted for age (y; continuous), sex (male or female, if applicable), smoking {smoking was defined as: 0 (never smokers); 1 [current light smokers (i.e., smoking <20 pack-years)]; 2 [current heavy smokers (i.e., smoking >20 pack-years)]; 3 [current smokers (no information on pack-years)]; 4 [former light smokers (i.e., smokers who ceased smoking >1 y prior and smoked <20 pack-years)]; 5 [former heavy smokers (i.e., smokers who ceased smoking >1 y prior and smoked >20 pack-years)]; or 6 [former smokers (smokers who ceased smoking >1 y prior and no information on pack-years)]}, total energy intake (kcal/d; continuous), ethnicity (Caucasian or non-Caucasian, if applicable), alcohol intake (mL/d; continuous), fruit intake (g/d; continuous), fat intake (g/d; continuous), meat intake (g/d; continuous), sugar intake (g/d; continuous), vegetable intake (g/d; continuous), and total fluid intake (mL/d; continuous).

#### Whole grain intake and BC risk

Table 2 shows the results of the Cox regression analyses for the associations between total whole grains and BC risk. In multivariable-adjusted analyses (model 2), higher total whole grain intake was significantly associated with lower BC risk (comparing the highest with the lowest tertile of intake: HR: 0.87; 95% CI: 0.77, 0.98; HR per 1-SD increment: 0.95; 95% CI: 0.91, 0.99; *P* for trend = 0.023). No evidence of association for tertile of intake was observed in the stratified analyses by cancer subtype, whereas the HR per 1-SD increment showed a borderline decreased risk (HR_model2_: 0.92; 95% CI: 0.85, 1.00; *P* for trend = 0.038) of MIBC. Results were consistent for both males (comparing the highest with the lowest intake tertile: HR_model2_: 0.85; 95% CI: 0.74, 0.98; HR per 1-SD increment: 0.93; 95% CI: 0.83, 1.02; *P* for trend = 0.059) and females (comparing the highest with the lowest intake tertile: HR_model2_: 0.83; 95% CI: 0.71, 0.96; HR per 1-SD increment: 0.93; 95% CI: 0.85, 1.01; *P* for trend = 0.053). No evidence of association was observed in the smoking-stratified analyses.

Of the individual whole grains assessed, only higher intake of brown rice was significantly associated with a decreased BC risk (comparing the highest with the lowest intake tertile: HR_model2_: 0.78; 95% CI: 0.67, 0.92; HR per 1-SD increment: 0.89; 95% CI: 0.82, 0.95; *P* for trend = 0.001) (**Table 3**). All other whole grains showed a null-association.

**TABLE 3:** Risk of bladder cancer according to individual intakes of whole grains and refined grains^1^

Grain source, subgroup, intake tertiles		Model 2^2^		
	Cases/participants, *n*	HR (95% CI)	HR per 1-SD increase (95% CI)	*P*-trend
Whole grains, g/d				
Brown rice				
Tertile 1	910/64,959	Ref.	0.89 (0.82, 0.95)	0.001
Tertile 2	262/64,685	0.97 (0.83, 1.13)		
Tertile 3	270/64,822	0.78 (0.67, 0.92)		
Wheat or oat				
Tertile 1	877/15,715	Ref.	0.99 (0.92, 1.06)	0.747
Tertile 2	81/3590	1.20 (0.95, 1.52)		
Tertile 3	210/9032	0.93 (0.80, 1.09)		
Basic products of other cereals^3^				
Tertile 1	820/4802	Ref.	0.98 (0.91, 1.06)	0.637
Tertile 2	25/233	0.78 (0.52, 1.16)		
Tertile 3	32/212	1.03 (0.72, 1.47)		
Refined grains, g/d				
White rice				
Tertile 1	976/44,980	Ref.	0.96 (0.88, 1.04)	0.344
Tertile 2	288/44,951	1.09 (0.93, 1.28)		
Tertile 3	221/44,954	1.05 (0.92, 1.21)		
Pasta or noodles				
Tertile 1	806/193,351	Ref.	0.99 (0.94, 1.04)	0.697
Tertile 2	787/188,377	0.90 (0.81, 0.99)		
Tertile 3	744/187,751	0.90 (0.80, 1.01)		
Leavened bread				
Tertile 1	1057/191,576	Ref.	0.99 (0.94, 1.05)	0.746
Tertile 2	1260/191,594	1.01 (0.91, 1.11)		
Tertile 3	897/191,556	1.01 (0.89, 1.15)		
Unleavened bread				
Tertile 1	775/119,122	Ref.	0.95 (0.89, 1.00)	0.070
Tertile 2	939/181,124	0.95 (0.85, 1.06)		
Tertile 3	863/181,124	0.97 (0.87, 1.09)		
Bakery wares				
Tertile 1	1732/477,213	Ref.	0.99 (0.96, 1.01)	0.307
Tertile 2	688/14,011	1.08 (0.76, 1.54)		
Tertile 3	448/14,011	1.00 (0.70, 1.44)		
Savory cereals dishes^4^				
Tertile 1	161/28,872	Ref.	0.95 (0.83, 1.08)	0.423
Tertile 2	96/18,996	0.96 (0.74, 1.24)		
Tertile 3	89/21,623	0.89 (0.67, 1.17)		
Breakfast cereals				
Tertile 1	1013/33,151	Ref.	0.97 (0.90, 1.04)	0.422
Tertile 2	251/32,949	1.01 (0.86, 1.20)		
Tertile 3	250/31,728	0.97 (0.81, 1.16)		

1 The intervals of tertiles were defined as follows: total whole grains: *1*) brown rice, 0 ≤ tertile 1 ≤ 4 g/d, 4 < tertile 2 ≤ 9 g/d, tertile 3 > 9 g/d; *2*) wheat or oat, tertile 1 = 0 g/d, 0 < tertile 2 ≤ 2 g/d, tertile 3 > 2 g/d; *3*) basic products of other cereals: tertile 1 = 0 g/d, 0 < tertile 2 ≤ 3 g/d, tertile 3 > 3 g/d; total refined grains: *1*) white rice, 0 ≤ tertile 1 ≤ 4 g/d, 4 < tertile 2 ≤ 11 g/d, tertile 3 > 11 g/d; *2*) pasta or noodles, 0 ≤ tertile 1 ≤ 3 g/d, 3 < tertile 2 ≤ 9 g/d, tertile 3 > 9 g/d; *3*) leavened bread, 0 ≤ tertile 1 ≤ 73 g/d, 73 < tertile 2 ≤ 160 g/d, tertile 3 > 160 g/d; *4*) unleavened bread, tertile 1 = 0 g/d, 0 < tertile 2 ≤ 4 g/d, tertile 3 > 4 g/d; *5*) bakery wares, tertile 1 = 0 g/d, 0 < tertile 2 ≤ 27 g/d, tertile 3 > 27 g/d; *6*) savory cereals dishes, 0 ≤ tertile 1 ≤ 3 g/d, 3 < tertile 2 ≤ 7 g/d, tertile 3 > 7 g/d; *7*) breakfast cereals, 0 ≤ tertile 1 ≤ 6 g/d, 6 < tertile 2 ≤ 27 g/d, tertile 3 > 27 g/d. Reference group was lowest intake (tertile 1). *P*-trend < 0.05 was considered statistically significant.

2 Model 2 of Cox regression: adjusted for age (y; continuous), sex (male/female), smoking {smoking was defined as: 0 (never smokers); 1 [current light smokers (i.e., smoking <20 pack-years)]; 2 [current heavy smokers (i.e., smoking >20 pack-years)]; 3 [current smokers (no information on pack-years)]; 4 [former light smokers (i.e., smokers who ceased smoking >1 y prior and smoked <20 pack-years)]; 5 [former heavy smokers (i.e., smokers who ceased smoking >1 y prior and smoked >20 pack-years)]; or 6 [former smokers (smokers who ceased smoking >1 y prior and no information on pack-years)]}, total energy intake (kcal/d; continuous), ethnicity (Caucasian or non-Caucasian, if applicable), alcohol intake (mL/d; continuous), fruit intake (g/d; continuous), fat intake (g/d; continuous), meat intake (g/d; continuous), sugar intake (g/d; continuous), vegetable intake (g/d; continuous), and total fluid intake (mL/d; continuous).

3 “Basic products of other cereals”: buckwheat, millet, sorghum, or spelt.

4 “Savory cereals dishes”: dumplings, couscous, risotto, pizza, pancake, or pie.

#### Refined grain intake and BC risk

Overall, no evidence of association between different categories of total refined grain intake and BC risk was observed. However, males showed a borderline decreased BC risk per 1-SD increment (HR_model 2_: 0.92; 95% CI: 0.86, 1.00; *P* for trend = 0.040) (Table 2). Looking at the individual refined grain sources, similar null-associations were found, except for the intake of “pasta or noodles,” which was inversely associated with BC risk when comparing medium intake with low intake (HR_model2_: 0.90; 95% CI: 0.81, 0.99; HR per 1-SD increment: 0.99; 95% CI: 0.94, 1.04; *P* for trend = 0.697) (Table 3).

#### Dietary fiber intake and BC risk

**Table 4** shows the associations of the intakes of total dietary fiber and dietary fiber from different food sources with BC risk. The intake of total dietary fiber was inversely associated with BC risk (comparing the highest with the lowest intake tertile: HR_model2_: 0.86; 95% CI: 0.76, 0.98; HR per 1-SD increment: 0.91; 95% CI: 0.82, 0.98; *P* for trend = 0.021). Consistent results were observed for both males (comparing the highest with the lowest intake tertile: HR_model2_: 0.89; 95% CI: 0.79, 0.98; HR per 1-SD increment: 0.90; 95% CI: 0.83, 0.97; *P* for trend = 0.007) and females (comparing the highest with the lowest intake tertile: HR_model2_: 0.79; 95% CI: 0.66, 0.97; HR per 1-SD increment: 0.89; 95% CI: 0.79, 1.00; *P* for trend = 0.049); however, no association was observed in the smoking-stratified analyses. For the individual dietary fiber food sources, only vegetable fiber showed a borderline decreased BC risk per 1-SD increment (HR_model2_: 0.93; 95% CI: 0.86, 1.00; *P* for trend = 0.046).

**TABLE 4:** Risk of bladder cancer according to intakes of total dietary fiber and individual sources of dietary fiber^1^

Grain source, subgroup, intake tertiles		Model 2^2^		
	Cases/participants, *n*	HR (95% CI)	HR per 1-SD increase (95% CI)	*P*-trend
Total dietary fiber, g/d				
Overall				
Tertile 1	1015/191,576	Ref.	0.91 (0.82, 0.98)	0.021
Tertile 2	1097/191,575	0.92 (0.83, 1.02)		
Tertile 3	1102/191,575	0.86 (0.76, 0.98)		
Male				
Tertile 1	775/62,954	Ref.	0.90 (0.83, 0.97)	0.007
Tertile 2	971/62,954	0.94 (0.85, 1.03)		
Tertile 3	670/62,954	0.89 (0.79, 0.98)		
Female				
Tertile 1	322/128,622	Ref.	0.89 (0.79, 1.00)	0.049
Tertile 2	272/128,621	0.81 (0.68, 0.96)		
Tertile 3	204/128,621	0.79 (0.66, 0.97)		
Never smoker				
Tertile 1	242/95,457	Ref.	0.95 (0.80, 1.12)	0.523
Tertile 2	272/95,457	0.91 (0.69, 1.20)		
Tertile 3	170/95,456	0.84 (0.67, 1.04)		
Current smoker				
Tertile 1	436/39,716	Ref.	0.94 (0.85, 1.03)	0.287
Tertile 2	438/39,715	0.92 (0.78, 1.09)		
Tertile 3	324/39,715	0.82 (0.67, 1.00)		
Former smoker				
Tertile 1	473/56,404	Ref.	0.89 (0.80, 1.00)	0.059
Tertile 2	492/56,403	0.97 (0.82, 1.14)		
Tertile 3	394/56,403	0.85 (0.70, 1.05)		
Cereal fiber, g/d				
Overall				
Tertile 1	1111/191,576	Ref.	0.96 (0.91, 1.01)	0.124
Tertile 2	1203/191,576	0.95 (0.86, 1.04)		
Tertile 3	900/191,574	0.95 (0.85, 1.07)		
Male				
Tertile 1	869/62,954	Ref.	0.91 (0.86, 1.01)	0.058
Tertile 2	1017/62,954	0.95 (0.85, 1.05)		
Tertile 3	530/62,954	0.89 (0.77, 1.03)		
Female				
Tertile 1	300/128,622	Ref.	0.97 (0.91, 1.07)	0.329
Tertile 2	293/128,621	1.07 (0.85, 1.34)		
Tertile 3	205/128,621	1.03 (0.86, 1.23)		
Never smoker				
Tertile 1	227/95,457	Ref.	0.98 (0.91, 1.04)	0.435
Tertile 2	245/95,457	1.01 (0.80, 1.34)		
Tertile 3	185/95,456	0.99 (0.81, 1.23)		
Current smoker				
Tertile 1	462/39,723	Ref.	0.97 (0.89, 1.06)	0.525
Tertile 2	416/39,718	1.00 (0.87, 1.16)		
Tertile 3	320/39,705	0.96 (0.79, 1.15)		
Former smoker				
Tertile 1	450/56,404	Ref.	0.90 (0.86, 1.01)	0.275
Tertile 2	557/56,403	0.88 (0.76, 1.02)		
Tertile 3	352/56,403	0.82 (0.69, 1.00)		
Fruit fiber, g/d				
Overall				
Tertile 1	1059/191,576	Ref.	0.98 (0.90, 1.06)	0.573
Tertile 2	950/191,613	0.98 (0.87, 1.11)		
Tertile 3	1205/191,537	0.97 (0.89, 1.07)		
Male				
Tertile 1	688/62,954	Ref.	1.01 (0.92, 1.11)	0.792
Tertile 2	689/62,954	0.98 (0.88, 1.10)		
Tertile 3	1039/62,954	1.02 (0.89, 1.17)		
Female				
Tertile 1	250/128,628	Ref.	0.87 (0.73, 1.03)	0.119
Tertile 2	264/128,615	0.94 (0.78, 1.13)		
Tertile 3	284/128,337	0.76 (0.58, 1.02)		
Never smoker				
Tertile 1	170/95,459	Ref.	0.94 (0.78, 1.13)	0.517
Tertile 2	231/95,455	1.02 (0.88, 1.19)		
Tertile 3	256/95,456	0.89 (0.73, 1.09)		
Current smoker				
Tertile 1	380/39,716	Ref.	0.99 (0.88, 1.18)	0.574
Tertile 2	376/39,715	1.17 (0.95, 1.43)		
Tertile 3	442/39,715	1.11 (0.83, 1.49)		
Former smoker				
Tertile 1	396/56,413	Ref.	0.97 (0.86, 1.10)	0.639
Tertile 2	384/56,394	0.93 (0.81, 1.08)		
Tertile 3	579/56,403	1.03 (0.86, 1.24)		
Vegetable fiber, g/d				
Overall				
Tertile 1	1185/191,576	Ref.	0.93 (0.86, 1.00)	0.046
Tertile 2	1223/191,575	0.98 (0.89, 1.08)		
Tertile 3	806/191,575	0.91 (0.79, 1.05)		
Male				
Tertile 1	718/62,954	Ref.	0.95 (0.88, 1.02)	0.176
Tertile 2	810/62,954	1.02 (0.91, 1.15)		
Tertile 3	888/62,954	0.90 (0.78, 1.05)		
Female				
Tertile 1	359/128,622	Ref.	0.88 (0.74, 1.05)	0.151
Tertile 2	295/128,621	0.90 (0.75, 1.08)		
Tertile 3	144/128,621	0.77 (0.59, 1.01)		
Never smoker				
Tertile 1	282/95,457	Ref.	0.92 (0.76, 1.12)	0.400
Tertile 2	237/95,457	0.93 (0.76, 1.13)		
Tertile 3	138/95,457	0.93 (0.70, 1.24)		
Current smoker				
Tertile 1	403/39,716	Ref.	0.87 (0.76, 1.01)	0.171
Tertile 2	453/39,715	0.95 (0.81, 1.12)		
Tertile 3	342/39,715	0.84 (0.67, 1.04)		
Former smoker				
Tertile 1	488/56,404	Ref.	0.94 (0.82, 1.07)	0.328
Tertile 2	497/56,403	1.01 (0.87, 1.17)		
Tertile 3	374/56,403	0.89 (0.72, 1.09)		

1 The intervals of tertiles were defined as follows: total dietary fiber: *1*) overall, 0 ≤ tertile 1 ≤ 17 g/d, 17 < tertile 2 ≤ 25 g/d, tertile 3 > 25 g/d; *2*) male, 0 ≤ tertile 1 ≤ 17 g/d, 17 < tertile 2 ≤ 26 g/d, tertile 3 > 26 g/d; *3*) female, 0 ≤ tertile 1 ≤ 18 g/d, 18 < tertile 2 ≤ 26 g/d, tertile 3 > 26 g/d; *4*) never smoker, 0 ≤ tertile 1 ≤ 18 g/d, 18 < tertile 2 ≤ 26 g/d, tertile 3 > 26 g/d; *5*) current smoker, 0 ≤ tertile 1 ≤ 17 g/d, 17 < tertile 2 ≤ 25 g/d, tertile 3 > 25 g/d; *6*) former smoker, 0 ≤ tertile 1 ≤ 17 g/d, 17 < tertile 2 ≤ 25 g/d, tertile 3 > 25 g/d; cereal fiber: *1*) overall, 0 ≤ tertile 1 ≤ 7 g/d, 7 < tertile 2 ≤ 12 g/d, tertile 3 > 12 g/d; *2*) male, 0 ≤ tertile 1 ≤ 7 g/d, 7 < tertile 2 ≤ 13 g/d, tertile 3 > 13 g/d; *3*) female, 0 ≤ tertile 1 ≤ 6 g/d, 6 < tertile 2 ≤ 11 g/d, tertile 3 > 11 g/d; *4*) never smoker, 0 ≤ tertile 1 ≤ 6 g/d, 6 < tertile 2 ≤ 11 g/d, tertile 3 > 11 g/d; *5*) current smoker, 0 ≤ tertile 1 ≤ 8 g/d, 8 < tertile 2 ≤ 13 g/d, tertile 3 > 13 g/d; *6*) former smoker, 0 ≤ tertile 1 ≤ 6 g/d, 6 < tertile 2 ≤ 11 g/d, tertile 3 > 11 g/d; fruit fiber: *1*) overall, 0 ≤ tertile 1 ≤ 2 g/d, 2 < tertile 2 ≤ 4 g/d, tertile 3 > 4 g/d; *2*) male, 0 ≤ tertile 1 ≤ 1 g/d, 1 < tertile 2 ≤ 3 g/d, tertile 3 > 3 g/d; *3*) female, 0 ≤ tertile 1 ≤ 2 g/d, 2 < tertile 2 ≤ 4 g/d, tertile 3 > 4 g/d; *4*) never smoker, 0 ≤ tertile 1 ≤ 2 g/d, 2 < tertile 2 ≤ 4 g/d, tertile 3 > 4 g/d; *5*) current smoker, 0 ≤ tertile 1 ≤ 1 g/d, 1 < tertile 2 ≤ 3 g/d, tertile 3 > 3 g/d; *6*) former smoker, 0 ≤ tertile 1 ≤ 1 g/d, 1 < tertile 2 ≤ 3 g/d, tertile 3 > 3 g/d; vegetable fiber: *1*) overall, 0 ≤ tertile 1 ≤ 5 g/d, 5 < tertile 2 ≤ 9 g/d, tertile 3 > 9 g/d; *2*) male, 0 ≤ tertile 1 ≤ 4 g/d, 4 < tertile 2 ≤ 7 g/d, tertile 3 > 7 g/d; *3*) female, 0 ≤ tertile 1 ≤ 5 g/d, 5 < tertile 2 ≤ 9 g/d, tertile 3 > 9 g/d; *4*) never smoker, 0 ≤ tertile 1 ≤ 5 g/d, 5 < tertile 2 ≤ 9 g/d, tertile 3 > 9 g/d; *5*) current smoker, 0 ≤ tertile 1 ≤ 5 g/d, 5 < tertile 2 ≤ 8 g/d, tertile 3 > 8 g/d; *6*) former smoker, 0 ≤ tertile 1 ≤ 4 g/d, 4 < tertile 2 ≤ 8 g/d, tertile 3 > 8 g/d. Reference group was lowest intake (tertile 1). *P*-trend < 0.05 was considered statistically significant.

2 Model 2 of Cox regression: adjusted for age (y; continuous), sex (male/female), smoking {smoking was defined as: 0 (never smokers); 1 [current light smokers (i.e., smoking <20 pack-years)]; 2 [current heavy smokers (i.e., smoking >20 pack-years)]; 3 [current smokers (no information on pack-years)]; 4 [former light smokers (i.e., smokers who ceased smoking >1 y prior and smoked <20 pack-years)]; 5 [former heavy smokers (i.e., smokers who ceased smoking >1 y prior and smoked >20 pack-years)]; or 6 [former smokers (smokers who ceased smoking >1 y prior and no information on pack-years)]}, total energy intake (kcal/d; continuous), ethnicity (Caucasian or non-Caucasian, if applicable), alcohol intake (mL/d; continuous), fruit intake (g/d; continuous), fat intake (g/d; continuous), meat intake (g/d; continuous), sugar intake (g/d; continuous), vegetable intake (g/d; continuous), and total fluid intake (mL/d; continuous).

#### Joint association of total whole grain and total dietary fiber with BC risk

**Table 5** shows the results of the Cox regression analyses for the potential joint effect of total whole grain and total dietary fiber on BC. Individuals with the highest intake of both total whole grain and total dietary fiber showed a 28% reduced BC risk (95% CI: 0.54, 0.93; *P* for trend = 0.031) compared with individuals with the lowest intakes of both total whole grain and total dietary fiber.

**TABLE 5:** Joint association of intake of total whole grain and total dietary fiber with bladder cancer risk^1^

	Total whole grain and total dietary fiber, g/d				
	Model 2^2^				
Total dietary fiber, g/d	Tertile 1	Tertile 2	Tertile 3	*P*-trend	*P*-interaction
Tertile 1					
Cases, *n*	348	421	222	0.031	0.027
HR (95% CI)	Ref.	0.91 (0.79, 1.06)	0.89 (0.70, 1.14)		
Tertile 2					
Cases, *n*	93	157	103		
HR (95% CI)	0.94 (0.74, 1.21)	0.86 (0.69, 1.08)	0.82 (0.63, 1.09)		
Tertile 3					
Cases, *n*	84	152	153		
HR (95% CI)	0.79 (0.60, 1.03)	0.71 (0.57, 0.88)	0.72 (0.54, 0.93)		

1 The intervals of tertiles were defined as follows: total whole grain: 0 ≤ tertile 1 ≤ 3 g/d, 3 < tertile 2 ≤ 8 g/d, tertile 3 > 8 g/d; total dietary fiber: 0 ≤ tertile 1 ≤ 17 g/d, 17 < tertile 2 ≤ 25 g/d, tertile 3 > 25 g/d. *P*-trend < 0.05 was considered statistically significant. Reference group was lowest intake of both total whole grain and total dietary fiber.

2 Model 2 of Cox regression: adjusted for age (y; continuous), sex (male/female), smoking {smoking was defined as: 0 (never smokers); 1 [current light smokers (i.e., smoking <20 pack-years)]; 2 [current heavy smokers (i.e., smoking >20 pack-years)]; 3 [current smokers (no information on pack-years)]; 4 [former light smokers (i.e., smokers who ceased smoking >1 y prior and smoked <20 pack-years)]; 5 [former heavy smokers (i.e., smokers who ceased smoking >1 y prior and smoked >20 pack-years)]; or 6 [former smokers (smokers who ceased smoking >1 y prior and no information on pack-years)]}, total energy intake (kcal/d; continuous), ethnicity (Caucasian or non-Caucasian, if applicable), alcohol intake (mL/d; continuous), fruit intake (g/d; continuous), fat intake (g/d; continuous), meat intake (g/d; continuous), sugar intake (g/d; continuous), vegetable intake (g/d; continuous), and total fluid intake (mL/d; continuous).

### Dose-response analyses

**FIGURE 1:**
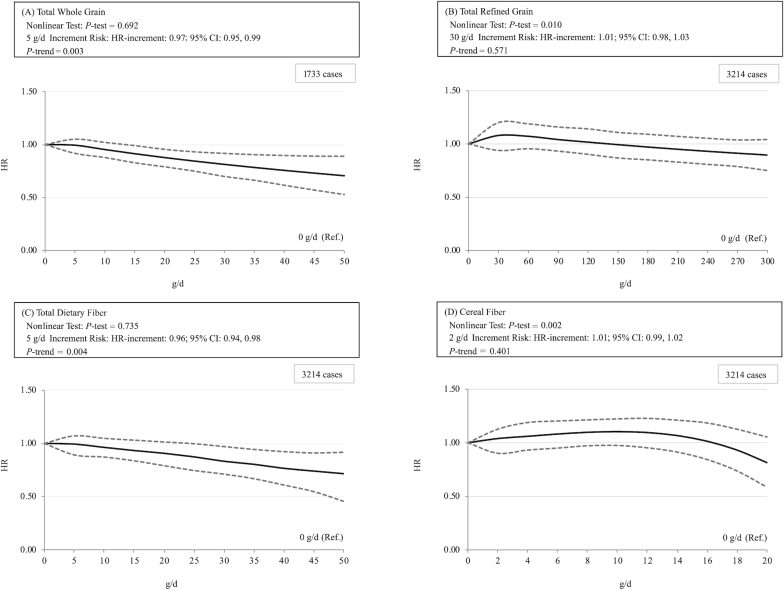
Dose–response relations between grain/dietary fiber intake and the risk of bladder cancer among total whole grain (A), total refined grain (B), total dietary fiber (C), and cereal fiber (D). The solid lines represent the HRs; the dashed lines represent the 95% CIs for the trend. The HRs were adjusted for age (y; continuous), sex (male/female), smoking {smoking was defined as: 0 (never smokers); 1 [current light smokers (i.e., smoking <20 pack-years)]; 2 [current heavy smokers (i.e., smoking >20 pack-years)]; 3 [current smokers (no information on pack-years)]; 4 [former light smokers (i.e., smokers who ceased smoking >1 y prior and smoked <20 pack-years)]; 5 [former heavy smokers (i.e., smokers who ceased smoking >1 y prior and smoked >20 pack-years)]; or 6 [former smokers (smokers who ceased smoking >1 y prior, and no information on pack-years)]}, ethnicity (Caucasian or non-Caucasian, if applicable), alcohol intake (mL/d; continuous), fruit intake (g/d; continuous), fat intake (g/d; continuous), meat intake (g/d; continuous), sugar intake (g/d; continuous), vegetable intake (g/d; continuous), and total fluid intake (mL/d; continuous) (model 2). Reference group was nonintake. *P* values < 0.05 were considered statistically significant for nonlinearity. *P*-increments < 0.05 were considered statistically significant.

**Figure 1**, **Supplemental Table 6**, and **Supplemental Figure 2** show dose–response relations between grain/dietary fiber intake and the risk of BC. There were inverse associations of intakes of total whole grain and total dietary fiber with BC risk, but no association was observed of intakes of total refined grain, cereal fiber, fruit fiber, and vegetable fiber with BC risk. A significant reduction of risk was shown at >15 g/d intake of total whole grain and >25 g/d intake of total dietary fiber; the estimated HRs were 0.97 (0.95, 0.99) and 0.96 (0.94, 0.98) per 5-g daily increment, respectively.

### Sensitivity analyses

Removing BC cases diagnosed within the first 2 y after enrolling into each individual study gave similar results, in which a decreased BC risk was observed for total whole grain (comparing the highest with the lowest intake tertile: HR_model2_: 0.81; 95% CI: 0.67, 0.97; HR per 1-SD increment: 0.95; 95% CI: 0.91, 1.00; *P* for trend = 0.040) and for total dietary fiber (comparing the highest with the lowest intake tertile: HR_model2_: 0.85; 95% CI: 0.76, 0.96; HR per 1-SD increment: 0.86; 95% CI: 0.78, 0.94; *P* for trend = 0.002) (**Supplemental Table 7**). The analysis excluding missing data (model 2) showed similar results to the analysis with multiple imputation (**Supplemental Table 8**). Results of the quintile-based analyses showed inverse associations between total whole grain and total dietary fiber intakes and BC risk (comparing the highest with the lowest intake quintile: HR_model2_: 0.86; 95% CI: 0.75, 0.98; *P* for trend = 0.032; and HR_model2_: 0.84; 95% CI: 0.72, 0.98; *P* for trend = 0.022, respectively) (**Supplemental Table 9**). In addition, excluding pasta as a source of total refined grain showed the same results as when pasta intake was included (**Supplemental Table 10**). The analysis adjusting for both smoking status and smoking pack-years showed similar results with the adjustment of the smoking dummy variable (**Supplemental Table 11**). The meta-analysis approach showed similar results, that is, a significantly reduced BC risk with total whole grain intake (HR: 0.89; 95% CI: 0.81, 0.98) and total dietary fiber intake (HR: 0.89; 95% CI: 0.80, 0.98), whereas there was no evidence of association for intakes of total refined grain and individual sources of dietary fiber with BC risk (**Supplementary Figure 3**); in addition, after removing the study that most likely dominated the analysis for each dietary factor, results remained the same (**Supplemental Table 12**).

## Discussion

This large, multicenter, prospective cohort study indicates a potential beneficial effect of total whole grain (particularly brown rice) and total dietary fiber intake for the prevention of BC, whereas intakes of total refined grain and individual fiber sources (i.e., cereal, fruit, and vegetable) did not show any significant association with BC risk.

To our knowledge, this is the first prospective study to investigate the association between whole grain intake and BC risk; in line with this result a previously conducted case-control study reported a nonsignificant protective effect of higher intake of total whole grains on BC risk (23), and another case-control study conducted in the 1980s reported a modest inverse association (24). Because it was reported that an increased BC risk was associated with a high dietary glycemic load (23), which has been reported to be reduced by the postprandial glucose and insulin responses to whole grain intake (61), the authors suggested that any potential benefit of whole grain intake to BC risk may act by mitigating the carcinogenic effects of hyperglycemia and hyperinsulinemia (62–64). In addition, our findings are strengthened by experimental studies which show that whole grains may exert their potential antitumor activity through the reduction of inflammation (65), which is thought to be related to a decreased BC risk (66).

One plausible reason total refined grain was not found to be protective for BC is that refined grains lack a high amount of dietary fiber and other bioactive compounds which are rich in whole grains (67, 68). The lack of dietary fiber could especially be emphasized because the present study shows that high intake both of total whole grain and of total dietary fiber was associated with a decreased BC risk. Epidemiological studies have shown that the intakes of whole grains and of dietary fiber were associated with a lower risk of chronic diseases (69–73), including various cancer types (74–76). In addition, experimental studies have reported that dietary fiber may protect cancer by improving insulin sensitivity and metabolic regulation, reducing inflammation, modulating the gut microbiota, removal of damaged cells, diluting carcinogens, and decreasing transit time (65, 77–81).

Although most standard pastas are made with refined wheat flour, several whole-wheat pastas are available and worldwide consumed in nontrivial amounts (68, 82). Considering that several cohorts have been conducted after the 2000s, the pasta intake could be a mixture of pasta made of refined and whole grains. Unfortunately, we were unable to distinguish between these types of pasta intake. Nevertheless, we only found a borderline-decreased BC risk for medium intake of “pasta or noodles.” In addition, the results of total refined grains were unchanged after excluding pasta intake. Because the lack of information on pasta types might have led to inaccurate estimates, results from the present study on the influence of pasta could be due to chance, therefore, they must be interpreted with caution. Further investigation of the influence of specific pasta types on BC risk may be warranted.

Half of the world's population relies on rice as a daily staple food and it is considered one of the most important crops globally. The association of this specific grain with BC risk might differ from any other grains, because rice has been reported to contain considerable amounts of inorganic arsenic (83), which has been acknowledged to be a human carcinogen for bladder (84). A previous prospective study in the United States found no association for BC risk with either brown rice or white rice (85). In our study we showed that both brown and white rice do not cause an increased BC risk; in addition, it has been reported that the washing and cooking procedure reduces the arsenic content of rice (83, 86), therefore, the potential influence of arsenic on the findings of the present study is minimal.

In the present study we found that high intake of total dietary fiber was significantly associated with a lower BC risk. However, none of the dietary fiber sources were associated with BC risk individually. One possible explanation is that individual dietary fiber intakes failed to reach the threshold at which a protective effect is manifested. In addition, some residual confounding such as “cooking methods” might mask the potential beneficial effect of cereal-, fruit-, or vegetable-fiber intake.

The joint effect of both whole grains and dietary fiber showed a stronger inverse association with BC risk than did the intakes individually. This finding confirmed the assumption that potential benefits of fiber may be derived from its combination with nutrients of whole grains working together, which suggests the simultaneous intake of both as part of a healthy, nutritious diet. Although current dietary guidelines recommend that individuals derive at least half of their grain intake from whole grains, no quantifications are given for the amount of total whole grains to be consumed (87). The present study shows that a daily intake of ≥15 g (uncooked) should be consumed in order to reduce the BC risk. For dietary fiber, the European Commission's strategy recommends a daily dietary fiber intake of 25–38 g in order to prevent noncommunicable diseases (88). This is in accordance with our observation of 25 g/d total dietary intake for reducing BC.

The present study pooled data from 13 prospective cohorts, enabling detailed analyses with good precision and statistical power. However, this study also includes some limitations: *1*) other than the included adjustments, limited information was available on other possible risk factors [e.g., BMI (89) and diabetes (90)] or dietary additives (e.g., dip or sauce); *2*) some tumor subtype information was missing, which hampered the statistical power required for stratified subgroup analyses; *3*) although status as well as duration and intensity of smoking were taken into account, residual confounding by smoking is difficult to rule out completely—however, because never smokers showed similar results to the overall analysis adjusted for smoking, the effect of residual confounding is likely to be minimal; *4*) people with a high intake of whole grains might have generally healthier lifestyles and diets than those with a low intake (91, 92), thus we could not rule out the possibility that some of the associations could be more likely due to a healthy lifestyle than to whole grain intake per se; *5*) the sample size for whole grains was significantly smaller than for refined grains and dietary fiber, which may have led to less statistical power; *6*) data were not available on dietary fiber types (e.g., soluble compared with insoluble), thus we were unable to investigate the association by dietary fiber subtypes; *7*) for most cohorts, the exposure variable was assessed by FFQs—therefore, measurement error and misclassification of study participants in terms of the exposure and outcome are unavoidable; and *8*) the present study sample consisted mostly of Caucasians, and this may limit the generalizability of our results to other racial/ethnic populations or geographic regions.

In summary, our study, of >3200 cases of incident BC occurring in almost 0.6 million participants, indicates a decreased BC risk with higher intakes of total whole grain (>8 g/d) and total dietary fiber (>23 g/d) individually and jointly. This, in turn, supports recommendations to increase the intakes of whole grains and dietary fiber to improve public health.

## Supplementary Material

nqaa215_Supplemental_File

## Notes

Supported in part by World Cancer Research Fund International grant WCRF 2012/590 (to MPZ) and European Commission grant FP7-PEOPLE-618308 (to MPZ). EYWY was supported by China Scholarship Council award no. 201706310135. The Netherlands Cohort Study on diet and cancer was supported by the Dutch Cancer Society. The RERF Atomic Bomb Survivors Study was supported by The Radiation Effects Research Foundation (RERF), Hiroshima and Nagasaki, Japan, a public interest foundation funded by the Japanese Ministry of Health, Labour and Welfare and the US Department of Energy (DOE). The research was also funded in part through DOE award DE-HS0000031 to the National Academy of Sciences. This publication was supported by RERF Research Protocol RP-A5-12. VITAL (VITamins and Lifestyle cohort study) was supported by National Cancer Institute grant R01CA74846. EPIC (The European Prospective Investigation into Cancer and Nutrition) was carried out with the financial support of the “Europe Against Cancer” Programme of the European Commission (SANCO); Ligue contre le Cancer (France); Société 3 M (France); Mutuelle Générale de l’Éducation Nationale; Institut National de la Santé et de la Recherche Médicale; Institute Gustave Roussy; German Cancer Aid; German Cancer Research Centre; German Federal Ministry of Education and Research; Danish Cancer Society; Health Research Fund (FIS) of the Spanish Ministry of Health; the Spanish Regional Governments of Andalucía, Asturias, Basque Country, Murcia, and Navarra; Cancer Research UK; Medical Research Council, UK; Stroke Association, UK; British Heart Foundation; Department of Health, UK; Food Standards Agency, UK; Wellcome Trust, UK; Greek Ministry of Health; Greek Ministry of Education; Italian Association for Research on Cancer; Italian National Research Council; Dutch Ministry of Public Health, Welfare and Sports; Dutch Prevention Funds; LK Research Funds; Dutch ZON (Zorg Onderzoek Nederland); World Cancer Research Fund; Swedish Cancer Society; Swedish Scientific Council; Regional Government of Skåne, Sweden; Norwegian Cancer Society; and Norwegian Research Council. Partial support for the publication of this supplement was provided by the Centre de Recherche et d'Information Nutritionnelles. Melbourne Collaborative Cohort Study (MCCS) cohort recruitment was funded by Cancer Council Victoria (http://www.cancervic.org.au/) and VicHealth (https://www.vichealth.vic.gov.au/). The MCCS was further augmented by Australian National Health and Medical Research Council grants 209057, 396414 and 1074383 and by infrastructure provided by Cancer Council Victoria. Cases and their vital status were ascertained through the Victorian Cancer Registry and the Australian Institute of Health and Welfare, including the National Death Index and the Australian Cancer Database.

Supplemental Figures 1–3 and Supplemental Tables 1–12 are available from the “Supplementary data” link in the online posting of the article and from the same link in the online table of contents at https://academic.oup.com/ajcn/.

The data that support the findings of this study are available on reasonable request pending approval from the corresponding author, AW. The data are not publicly available owing to their containing information that could compromise the privacy of research participants.

Abbreviations used: BC, bladder cancer; BLEND, BLadder cancer Epidemiology and Nutritional Determinants; MIBC, muscle invasive bladder cancer; NLCS, NetherLands Cohort Study; NMIBC, nonmuscle invasive bladder cancer.

## References

[1] Siegel RL, Miller KD, Jemal A Cancer statistics, 2019. CA Cancer J Clin. 2019;69(1):7–34. 30620402 10.3322/caac.21551

[2] Richters A, Aben KKH, Kiemeney LALM The global burden of urinary bladder cancer: an update. World J Urol. 2020;38(8):1895–904. 31676912 10.1007/s00345-019-02984-4 PMC7363726

[3] Antoni S, Ferlay J, Soerjomataram I, Znaor A, Jemal A, Bray F Bladder cancer incidence and mortality: a global overview and recent trends. Eur Urol. 2017;71(1):96–108. 27370177 10.1016/j.eururo.2016.06.010

[4] Jemal A, Bray F, Center MM, Ferlay J, Ward E, Forman D Global cancer statistics. CA Cancer J Clin. 2011;61(2):69–90. 21296855 10.3322/caac.20107

[5] Ploeg M, Aben KK, Kiemeney LA The present and future burden of urinary bladder cancer in the world. World J Urol. 2009;27(3):289–93. 19219610 10.1007/s00345-009-0383-3 PMC2694323

[6] Marugame T, Mizuno S Comparison of prostate cancer mortality in five countries: France, Italy, Japan, UK and USA from the WHO mortality database (1960–2000). Jpn J Clin Oncol. 2005;35(11):690–1. 16306486 10.1093/jjco/hyi185

[7] Stewart B, Wild CP World cancer report 2014. Geneva, Switzerland: WHO Press; 2014.

[8] Letašiová S, Medve'ová A, Šovčíková A, Dušinská M, Volkovová K, Mosoiu C, Bartonová A Bladder cancer, a review of the environmental risk factors. Environ Health. 2012;11(Suppl 1):S11. 22759493 10.1186/1476-069X-11-S1-S11 PMC3388449

[9] Botteman MF, Pashos CL, Redaelli A, Laskin B, Hauser R The health economics of bladder cancer: a comprehensive review of the published literature. Pharmacoeconomics. 2003;21(18):1315–30. 14750899 10.1007/BF03262330

[10] Al-Zalabani AH, Stewart KF, Wesselius A, Schols AM, Zeegers MP Modifiable risk factors for the prevention of bladder cancer: a systematic review of meta-analyses. Eur J Epidemiol. 2016;31(9):811–51. 27000312 10.1007/s10654-016-0138-6 PMC5010611

[11] Piyathilake C Dietary factors associated with bladder cancer. Investig Clin Urol. 2016;57(Suppl 1):S14–25. 10.4111/icu.2016.57.S1.S14 PMC4910759 27326403

[12] Johansson SL, Cohen SM Epidemiology and etiology of bladder cancer. Semin Surg Oncol. 1997;13(5):291–8. 9259084 10.1002/(sici)1098-2388(199709/10)13:5<291::aid-ssu2>3.0.co;2-8

[13] Thun M, Linet MS, Cerhan JR, Haiman CA, Schottenfeld D Cancer epidemiology and prevention. 4th ed Oxford: Oxford University Press; 2017.

[14] Oberoi S, Barchowsky A, Wu F The global burden of disease for skin, lung, and bladder cancer caused by arsenic in food. Cancer Epidemiol Biomarkers Prev. 2014;23(7):1187–94. 24793955 10.1158/1055-9965.EPI-13-1317 PMC4082465

[15] European Food Information Council (EUFIC) [Internet] Brussels, Belgium: EUFIC; 2015 [cited July 15, 2019] Available from: https://www.eufic.org/en/whats-in-food/article/whole-grains-updated-2015.

[16] US Department of Agriculture and US Department of Health and Human Services Dietary guidelines for Americans. [Internet] Alexandria, VA: USDA Center for Nutrition Policy & Promotion; 2018 [cited July 15, 2019] Available from: https://www.choosemyplate.gov/dietary-guidelines.

[17] Mozaffarian RS, Lee RM, Kennedy MA, Ludwig DS, Mozaffarian D, Gortmaker SL Identifying whole grain foods: a comparison of different approaches for selecting more healthful whole grain products. Public Health Nutr. 2013;16(12):2255–64. 23286205 10.1017/S1368980012005447 PMC4486284

[18] US Department of Health and Human Services (USDHHS) and USDA 2015–2020 Dietary Guidelines for Americans. [Internet] 8th ed Washington (DC): USDHHS and USDA; December 2015; . [cited September 7, 2019]. Available from: http://health.gov/dietaryguidelines/2015/guidelines/.

[19] Makarem N, Nicholson JM, Bandera EV, McKeown NM, Parekh N Consumption of whole grains and cereal fiber in relation to cancer risk: a systematic review of longitudinal studies. Nutr Rev. 2016;74(6):353–73. 27257283 10.1093/nutrit/nuw003 PMC4892300

[20] Hsu IR, Kim SP, Kabir M, Bergman RN Metabolic syndrome, hyperinsulinemia, and cancer. Am J Clin Nutr. 2007;86(3):s867–71. 18265480 10.1093/ajcn/86.3.867S

[21] Murata M Inflammation and cancer. Environ Health Prev Med. 2018;23(1):50. 30340457 10.1186/s12199-018-0740-1 PMC6195709

[22] Anderson JW, Baird P, Davis RH Jr, Ferreri S, Knudtson M, Koraym A, Waters V, Williams CL Health benefits of dietary fiber. Nutr Rev. 2009;67(4):188–205. 19335713 10.1111/j.1753-4887.2009.00189.x

[23] Augustin LSA, Taborelli M, Montella M, Libra M, La Vecchia C, Tavani A, Crispo A, Grimaldi M, Facchini G, Jenkins DJA et al. Associations of dietary carbohydrates, glycaemic index and glycaemic load with risk of bladder cancer: a case-control study. Br J Nutr. 2017;118(9):722–9. 28990544 10.1017/S0007114517002574

[24] La Vecchia C, Chatenoud L, Negri E, Franceschi S Wholegrain cereals and cancer in Italy. Proc Nutr Soc. 2003;62(1):45–9. 12740056 10.1079/PNS2002235

[25] Nicodemus KK, Jacobs DR Jr, Folsom AR Whole and refined grain intake and risk of incident postmenopausal breast cancer (United States). Cancer Causes Control. 2001;12(10):917–25. 11808711 10.1023/a:1013746719385

[26] Farvid MS, Cho E, Eliassen AH, Chen WY, Willett WC Lifetime grain consumption and breast cancer risk. Breast Cancer Res Treat. 2016;159(2):335–45. 27510186 10.1007/s10549-016-3910-0 PMC5014619

[27] Kasum CM, Jacobs DR Jr, Nicodemus K, Folsom AR Dietary risk factors for upper aerodigestive tract cancers. Int J Cancer. 2002;99(2):267–72. 11979443 10.1002/ijc.10341

[28] Kasum CM, Nicodemus K, Harnack LJ, Jacobs DR Jr, Folsom AR Whole grain intake and incident endometrial cancer: the Iowa Women's Health Study. Nutr Cancer. 2001;39(2):180–6. 11759278 10.1207/S15327914nc392_4

[29] Makarem N, Bandera EV, Lin Y, McKeown NM, Hayes RB, Parekh N Associations of whole and refined grain intakes with adiposity-related cancer risk in the Framingham Offspring Cohort (1991–2013). Nutr Cancer. 2018;70(5):776–86. 29781707 10.1080/01635581.2018.1470647 PMC7236605

[30] Xu Y, Yang J, Du L, Li K, Zhou Y Association of whole grain, refined grain, and cereal consumption with gastric cancer risk: a meta-analysis of observational studies. Food Sci Nutr. 2019;7(1):256–65. 30680179 10.1002/fsn3.878 PMC6341150

[31] Schwingshackl L, Schwedhelm C, Hoffmann G, Knuppel S, Laure Preterre A, Iqbal K, Bechthold A, De Henauw S, Michels N, Devleesschauwer B et al. Food groups and risk of colorectal cancer. Int J Cancer. 2018;142(9):1748–58. 29210053 10.1002/ijc.31198

[32] Riboli E, Kaaks R The EPIC Project: rationale and study design. European Prospective Investigation into Cancer and Nutrition. Int J Epidemiol. 1997;26(Suppl 1):S6–14. 9126529 10.1093/ije/26.suppl_1.s6

[33] Tjønneland A, Olsen A, Boll K, Stripp C, Christensen J, Engholm G, Overvad K Study design, exposure variables, and socioeconomic determinants of participation in Diet, Cancer and Health: a population-based prospective cohort study of 57,053 men and women in Denmark. Scand J Public Health. 2007;35(4):432–41. 17786808 10.1080/14034940601047986

[34] Clavel-Chapelon F, van Liere MJ, Giubout C, Niravong MY, Goulard H, Le Corre C, Hoang LA, Amoyel J, Auquier A, Duquesnel E E3N, a French cohort study on cancer risk factors. E3N Group. Etude Epidémiologique auprès de femmes de l'Education Nationale. Eur J Cancer Prev. 1997;6(5):473–8. 9466118 10.1097/00008469-199710000-00007

[35] Boeing H, Korfmann A, Bergmann MM Recruitment procedures of EPIC-Germany. European Investigation into Cancer and Nutrition. Ann Nutr Metab. 1999;43(4):205–15. 10592369 10.1159/000012787

[36] Riboli E, Hunt KJ, Slimani N, Ferrari P, Norat T, Fahey M, Charrondière UR, Hémon B, Casagrande C, Vignat J et al. European Prospective Investigation into Cancer and Nutrition (EPIC): study populations and data collection. Public Health Nutr. 2002;5(6b):1113–24. 12639222 10.1079/PHN2002394

[37] Panico S, Dello Iacovo R, Celentano E, Galasso R, Muti P, Salvatore M, Mancini M Progetto ATENA, a study on the etiology of major chronic diseases in women: design, rationale and objectives. Eur J Epidemiol. 1992;8(4):601–8. 1397231 10.1007/BF00146383

[38] Manjer J, Carlsson S, Elmståhl S, Gullberg B, Janzon L, Lindström M, Mattisson I, Berglund G The Malmö Diet and Cancer Study: representativity, cancer incidence and mortality in participants and non-participants. Eur J Cancer Prev. 2001;10(6):489–99. 11916347 10.1097/00008469-200112000-00003

[39] Hallmans G, Ågren Å, Johansson G, Johansson A, Stegmayr B, Jansson J-H, Lindahl B, Rolandsson O, Söderberg S, Nilsson M et al. Cardiovascular disease and diabetes in the Northern Sweden Health and Disease Study Cohort - evaluation of risk factors and their interactions. Scand J Public Health Suppl. 2003;61:18–24. 14660243 10.1080/14034950310001432

[40] Beulens JW, Monninkhof EM, Verschuren WM, van der Schouw YT, Smit J, Ocke MC, Jansen EH, van Dieren S, Grobbee DE, Peeters PH et al. Cohort profile: the EPIC-NL study. Int J Epidemiol. 2010;39(5):1170–8. 19483199 10.1093/ije/dyp217

[41] Davey GK, Spencer EA, Appleby PN, Allen NE, Knox KH, Key TJ EPIC-Oxford: lifestyle characteristics and nutrient intakes in a cohort of 33 883 meat-eaters and 31 546 non meat-eaters in the UK. Public Health Nutr. 2003;6(3):259–69. 12740075 10.1079/PHN2002430

[42] Day N, Oakes S, Luben R, Khaw KT, Bingham S, Welch A, Wareham N EPIC-Norfolk: study design and characteristics of the cohort. European Prospective Investigation of Cancer. Br J Cancer. 1999;80(Suppl 1):95–103. 10466767

[43] Lund E, Dumeaux V, Braaten T, Hjartåker A, Engeset D, Skeie G, Kumle M Cohort profile: The Norwegian Women and Cancer Study—NOWAC—Kvinner og kreft. Int J Epidemiol. 2008;37(1):36–41. 17644530 10.1093/ije/dym137

[44] van den Brandt PA, Goldbohm RA, van ’t Veer P, Volovics A, Hermus RJ, Sturmans F A large-scale prospective cohort study on diet and cancer in The Netherlands. J Clin Epidemiol. 1990;43(3):285–95. 2313318 10.1016/0895-4356(90)90009-e

[45] White E, Patterson RE, Kristal AR, Thornquist M, King I, Shattuck AL, Evans I, Satia-Abouta J, Littman AJ, Potter JD VITamins And Lifestyle cohort study: study design and characteristics of supplement users. Am J Epidemiol. 2004;159(1):83–93. 14693663 10.1093/aje/kwh010

[46] Giles GG, English DR The Melbourne Collaborative Cohort Study. IARC Sci Publ. 2002;156:69–70. 12484128

[47] Milne RL, Fletcher AS, MacInnis RJ, Hodge AM, Hopkins AH, Bassett JK, Bruinsma FJ, Lynch BM, Dugue PA, Jayasekara H et al. Cohort profile: the Melbourne Collaborative Cohort Study (Health 2020). Int J Epidemiol. 2017;46(6):1757–7i. 28641380 10.1093/ije/dyx085

[48] Goossens ME, Isa F, Brinkman M, Mak D, Reulen R, Wesselius A, Benhamou S, Bosetti C, Bueno-de-Mesquita B, Carta A et al. International pooled study on diet and bladder cancer: the bladder cancer, epidemiology and nutritional determinants (BLEND) study: design and baseline characteristics. Arch Public Health. 2016;74:30. 27386115 10.1186/s13690-016-0140-1 PMC4933992

[49] Bassett JK, English DR, Fahey MT, Forbes AB, Gurrin LC, Simpson JA, Brinkman MT, Giles GG, Hodge AM Validity and calibration of the FFQ used in the Melbourne Collaborative Cohort Study. Public Health Nutr. 2016;19(13):2357–68. 27075344 10.1017/S1368980016000690 PMC10271053

[50] Hodge A, Patterson AJ, Brown WJ, Ireland P, Giles G The Anti Cancer Council of Victoria FFQ: relative validity of nutrient intakes compared with weighed food records in young to middle-aged women in a study of iron supplementation. Aust N Z J Public Health. 2000;24(6):576–83. 11215004 10.1111/j.1467-842x.2000.tb00520.x

[51] Kaaks R, Riboli E Validation and calibration of dietary intake measurements in the EPIC project: methodological considerations. European Prospective Investigation into Cancer and Nutrition. Int J Epidemiol. 1997;26(Suppl 1):S15–25. 9126530 10.1093/ije/26.suppl_1.s15

[52] Zeegers MP, Goldbohm RA, van den Brandt PA Are retinol, vitamin C, vitamin E, folate and carotenoids intake associated with bladder cancer risk? Results from the Netherlands Cohort Study. Br J Cancer. 2001;85(7):977–83. 11592769 10.1054/bjoc.2001.1968 PMC2375109

[53] Ferrari P, Slimani N, Ciampi A, Trichopoulou A, Naska A, Lauria C, Veglia F, Bueno-de-Mesquita HB, Ocke MC, Brustad M et al. Evaluation of under- and overreporting of energy intake in the 24-hour diet recalls in the European Prospective Investigation into Cancer and Nutrition (EPIC). Public Health Nutr. 2002;5(6b):1329–45. 12639236 10.1079/PHN2002409

[54] Poortvliet E, Klensin J, Kohlmeier L Rationale document for the Eurocode 2 food coding system (version 91/2). Eur J Clin Nutr. 1992;46(Suppl 5):S9–S24. 1486874

[55] Watt BK, Merrill AL, Orr ML, Wu W, Pecot R Composition of foods: raw, processed, prepared. Washington (DC): Consumer and Food Economics Institute, Agricultural Research Service, USDA; 1964.

[56] Giganti MJ, Luz PM, Caro-Vega Y, Cesar C, Padgett D, Koenig S, Echevarria J, McGowan CC, Shepherd BE A comparison of seven Cox regression-based models to account for heterogeneity across multiple HIV treatment cohorts in Latin America and the Caribbean. AIDS Res Hum Retroviruses. 2015;31(5):496–503. 25647087 10.1089/aid.2014.0241 PMC4426314

[57] Willett WC, Howe GR, Kushi LH Adjustment for total energy intake in epidemiologic studies. Am J Clin Nutr. 1997;65(4 Suppl):1220S–8S.; discussion 1229S–31S. 9094926 10.1093/ajcn/65.4.1220S

[58] Bagnardi V, Zambon A, Quatto P, Corrao G Flexible meta-regression functions for modeling aggregate dose-response data, with an application to alcohol and mortality. Am J Epidemiol. 2004;159(11):1077–86. 15155292 10.1093/aje/kwh142

[59] Royston P, Altman DG Approximating statistical functions by using fractional polynomial regression. J R Stat Soc Ser D Stat. 1997;46(3):411–22.

[60] Jones BL, Nagin DS A Stata plugin for estimating group-based trajectory models. [Internet] Research Showcase@ CMU Carnegie Mellon University Bloomington, IN: Indiana University Bloomington Social Science Research Commons; 2012; [cited July 10, 2015] Available from: https://ssrc.indiana.edu/doc/wimdocs/2013-03-29_nagin_trajectory_stata-plugin-info.pdf.

[61] Esmaillzadeh A, Mirmiran P, Azizi F Whole-grain consumption and the metabolic syndrome: a favorable association in Tehranian adults. Eur J Clin Nutr. 2005;59(3):353–62. 15536473 10.1038/sj.ejcn.1602080

[62] Jee SH, Ohrr H, Sull JW, Yun JE, Ji M, Samet JM Fasting serum glucose level and cancer risk in Korean men and women. JAMA. 2005;293(2):194–202. 15644546 10.1001/jama.293.2.194

[63] Liu S, Li Y, Lin T, Fan X, Liang Y, Heemann U High dose human insulin and insulin glargine promote T24 bladder cancer cell proliferation via PI3K-independent activation of Akt. Diabetes Res Clin Pract. 2011;91(2):177–82. 21129803 10.1016/j.diabres.2010.11.009

[64] Ornskov D, Nexo E, Sorensen BS Insulin-induced proliferation of bladder cancer cells is mediated through activation of the epidermal growth factor system. FEBS J. 2006;273(23):5479–89. 17116246 10.1111/j.1742-4658.2006.05539.x

[65] Qi L, van Dam RM, Liu S, Franz M, Mantzoros C, Hu FB Whole-grain, bran, and cereal fiber intakes and markers of systemic inflammation in diabetic women. Diabetes Care. 2006;29(2):207–11. 16443861 10.2337/diacare.29.02.06.dc05-1903

[66] Gakis G The role of inflammation in bladder cancer. Adv Exp Med Biol. 2014;816:183–96. 24818724 10.1007/978-3-0348-0837-8_8

[67] Slavin J Why whole grains are protective: biological mechanisms. Proc Nutr Soc. 2003;62(1):129–34. 12740067 10.1079/PNS2002221

[68] Frolich W, Aman P, Tetens I Whole grain foods and health – a Scandinavian perspective. Food Nutr Res. 2013;57:18503. 10.3402/fnr.v57i0.18503 PMC3572214 23411562

[69] de Munter JS, Hu FB, Spiegelman D, Franz M, van Dam RM Whole grain, bran, and germ intake and risk of type 2 diabetes: a prospective cohort study and systematic review. PLoS Med. 2007;4(8):e261. 17760498 10.1371/journal.pmed.0040261 PMC1952203

[70] Ye EQ, Chacko SA, Chou EL, Kugizaki M, Liu S Greater whole-grain intake is associated with lower risk of type 2 diabetes, cardiovascular disease, and weight gain. J Nutr. 2012;142(7):1304–13. 22649266 10.3945/jn.111.155325 PMC6498460

[71] Cho SS, Qi L, Fahey GC Jr, Klurfeld DM Consumption of cereal fiber, mixtures of whole grains and bran, and whole grains and risk reduction in type 2 diabetes, obesity, and cardiovascular disease. Am J Clin Nutr. 2013;98(2):594–619. 23803885 10.3945/ajcn.113.067629

[72] Steffen LM, Jacobs DR Jr, Murtaugh MA, Moran A, Steinberger J, Hong CP, Sinaiko AR Whole grain intake is associated with lower body mass and greater insulin sensitivity among adolescents. Am J Epidemiol. 2003;158(3):243–50. 12882946 10.1093/aje/kwg146

[73] Mellen PB, Walsh TF, Herrington DM Whole grain intake and cardiovascular disease: a meta-analysis. Nutr Metab Cardiovasc Dis. 2008;18(4):283–90. 17449231 10.1016/j.numecd.2006.12.008

[74] Yang W, Ma Y, Liu Y, Smith-Warner SA, Simon TG, Chong DQ, Qi Q, Meyerhardt JA, Giovannucci EL, Chan AT et al. Association of intake of whole grains and dietary fiber with risk of hepatocellular carcinoma in US adults. JAMA Oncol. 2019;5(6):879–86. 30789662 10.1001/jamaoncol.2018.7159 PMC6567825

[75] Skeie G, Braaten T, Olsen A, Kyrø C, Tjønneland A, Landberg R, Nilsson LM, Wennberg M, Overvad K, Åsli LA et al. Intake of whole grains and incidence of oesophageal cancer in the HELGA Cohort. Eur J Epidemiol. 2016;31(4):405–14. 26092139 10.1007/s10654-015-0057-y

[76] Lei Q, Zheng H, Bi J, Wang X, Jiang T, Gao X, Tian F, Xu M, Wu C, Zhang L et al. Whole grain intake reduces pancreatic cancer risk: a meta-analysis of observational studies. Medicine (Baltimore). 2016;95(9):e2747. 26945361 10.1097/MD.0000000000002747 PMC4782845

[77] Te Morenga L, Docherty P, Williams S, Mann J The effect of a diet moderately high in protein and fiber on insulin sensitivity measured using the Dynamic Insulin Sensitivity and Secretion Test (DISST). Nutrients. 2017;9(12):1291. 10.3390/nu9121291 PMC5748742 29186908

[78] Kasubuchi M, Hasegawa S, Hiramatsu T, Ichimura A, Kimura I Dietary gut microbial metabolites, short-chain fatty acids, and host metabolic regulation. Nutrients. 2015;7(4):2839–49. 25875123 10.3390/nu7042839 PMC4425176

[79] Hemarajata P, Versalovic J Effects of probiotics on gut microbiota: mechanisms of intestinal immunomodulation and neuromodulation. Therap Adv Gastroenterol. 2013;6(1):39–51. 10.1177/1756283X12459294 PMC3539293 23320049

[80] Aron-Wisnewsky J, Clement K The gut microbiome, diet, and links to cardiometabolic and chronic disorders. Nat Rev Nephrol. 2016;12(3):169–81. 26616538 10.1038/nrneph.2015.191

[81] Aune D, Keum N, Giovannucci E, Fadnes LT, Boffetta P, Greenwood DC, Tonstad S, Vatten LJ, Riboli E, Norat T Whole grain consumption and risk of cardiovascular disease, cancer, and all cause and cause specific mortality: systematic review and dose-response meta-analysis of prospective studies. BMJ. 2016;353:i2716. 27301975 10.1136/bmj.i2716 PMC4908315

[82] Chatenoud L, Tavani A, La Vecchia C, Jacobs DR Jr, Negri E, Levi F, Franceschi S Whole grain food intake and cancer risk. Int J Cancer. 1998;77(1):24–8. 9639389 10.1002/(sici)1097-0215(19980703)77:1<24::aid-ijc5>3.0.co;2-1

[83] Kumarathilaka P, Seneweera S, Ok YS, Meharg A, Bundschuh J Arsenic in cooked rice foods: assessing health risks and mitigation options. Environ Int. 2019;127:584–91. 30986740 10.1016/j.envint.2019.04.004

[84] ARC Arsenic, metals, fibers and dusts: a review of human carcinogens. IARC Monogr Eval Carcinog Risks Hum. 2012;100C:1–527. PMC4781271 23189751

[85] Zhang R, Zhang X, Wu K, Wu H, Sun Q, Hu FB, Han J, Willett WC, Giovannucci EL Rice consumption and cancer incidence in US men and women. Int J Cancer. 2016;138(3):555–64. 26219234 10.1002/ijc.29704 PMC4919813

[86] Althobiti RA, Sadiq NW, Beauchemin D Realistic risk assessment of arsenic in rice. Food Chem. 2018;257:230–6. 29622204 10.1016/j.foodchem.2018.03.015

[87] Marmot M, Atinmo T, Byers T, Chen J, Hirohata T, Jackson A, James W, Kolonel L, Kumanyika S, Leitzmann C Food, nutrition, physical activity, and the prevention of cancer: a global perspective. Washington (DC): American Institute for Cancer Research; 2007.

[88] European Commission Health promotion and disease prevention knowledge gateway: dietary fibre. [Internet] Ispra, Italy: Joint Research Center; 2011 [cited September 17, 2019]. Available from: https://ec.europa.eu/jrc/en/health-knowledge-gateway/promotion-prevention/nutrition/fibre.

[89] Sun J-W, Zhao L-G, Yang Y, Ma X, Wang Y-Y, Xiang Y-B Obesity and risk of bladder cancer: a dose-response meta-analysis of 15 cohort studies. PLoS One. 2015;10(3):e0119313. 25803438 10.1371/journal.pone.0119313 PMC4372289

[90] Xu Y, Huo R, Chen X, Yu X Diabetes mellitus and the risk of bladder cancer: a PRISMA-compliant meta-analysis of cohort studies. Medicine (Baltimore). 2017;96(46):e8588. 29145273 10.1097/MD.0000000000008588 PMC5704818

[91] Kyrø C, Skeie G, Dragsted LO, Christensen J, Overvad K, Hallmans G, Johansson I, Lund E, Slimani N, Johnsen NF et al. Intake of whole grains in Scandinavia is associated with healthy lifestyle, socio-economic and dietary factors. Public Health Nutr. 2011;14(10):1787–95. 21338557 10.1017/S1368980011000206

[92] Wu H, Flint AJ, Qi Q, van Dam RM, Sampson LA, Rimm EB, Holmes MD, Willett WC, Hu FB, Sun Q Association between dietary whole grain intake and risk of mortality: two large prospective studies in US men and women. JAMA Intern Med. 2015;175(3):373–84. 25559238 10.1001/jamainternmed.2014.6283 PMC4429593

