# Supplementary material for: Grain and dietary fiber intake and bladder cancer risk: a pooled analysis of prospective cohort studies
Source: Am J Clin Nutr. 2020 Aug 10;112(5):1252–66. doi: 10.1093/ajcn/nqaa215 (PMC7657329; doi:10.1093/ajcn/nqaa215)
Supplement: nqaa215_Supplemental_File [file nqaa215_supplemental_file.pdf]

Supplementary Material for:

Grains and dietary fiber intake and bladder cancer risk: a pooled analysis of prospective cohort studies

Evan Y.W. Yu, Anke Wesselius $^{\Psi}$, Siamak Mehrkanoon, Maree Brinkman, Piet van den Brandt, Emily White, Elisabete Weiderpass, Florence Le Calvez-Kelm, Marc Gunter, Inge Huybrechts, Fredrik Liedberg, Guri Skeie, Anne Tjonneland, Elio Riboli, Graham G. Giles, Roger L. Milne, Maurice P. Zeegers

$^{\Psi}$to whom correspondence should be addressed: anke.wesselius@maastrichtuniversity.nl

Grains and dietary fiber intake and bladder cancer risk: a pooled analysis of prospective cohort studies
Yu et. al
On-line Supplementary Material

Supplementary Figure 1 Flow diagram of participants included in the statistical analyses on grain and dietary fiber intake and bladder cancer risk

Supplementary Table 1 Additional baseline characteristic of the participant cohort studies

Supplementary Table 2 Risk of bladder cancer according to intake of total grain, total whole grain and total refined grain (model 1)

Supplementary Table 3 Risk of bladder cancer according to individual intake of whole grains and refined grains (model 1)

Supplementary Table 4 Risk of bladder cancer according to intake of total dietary fiber and individual source of dietary fiber (model 1)

Supplementary Table 5 Joint association of intake of total whole grain and total dietary fiber with bladder cancer risk (model 1)

Supplementary Table 6 Risk of bladder cancer of each dose-response relationship according to intakes of grain and dietary fiber (model 2)

Supplementary Table 7 Risk of bladder cancer according to grains and dietary fiber intake (after removing 358 cases diagnosed within 2 years)

Supplementary Table 8 Risk of bladder cancer according to intakes of grain and dietary fiber based on complete dataset (model 2)

Supplementary Table 9 Risk of bladder cancer according to intakes of grain and dietary fiber based on quintile analyses

Supplementary Figure 2 Dose-response relationships between dietary fiber intake and the risk of bladder cancer among (A) fruit fiber and (B) vegetable fiber

Supplementary Table 10 Risk of bladder cancer risk according to intake of total refined grain (after removing pasta intake)

Supplementary Table 11 Risk of bladder cancer according to intake of total whole grain, total refined grain and total dietary fiber in different adjustment models

Supplementary Figure 3 Forest plot of meta-analyses with HRs and 95% CIs for highest vs. lowest intake of grain and dietary fiber with bladder cancer risk on (A) total whole grain; (B) total refined grain; (C) total dietary fiber; (D) cereal fiber; (E) fruit fiber; (F) vegetable fiber

Supplementary Table 12 Risk of bladder cancer according to intakes of grain and dietary fiber after removing the study that most likely dominates the results (model 2)

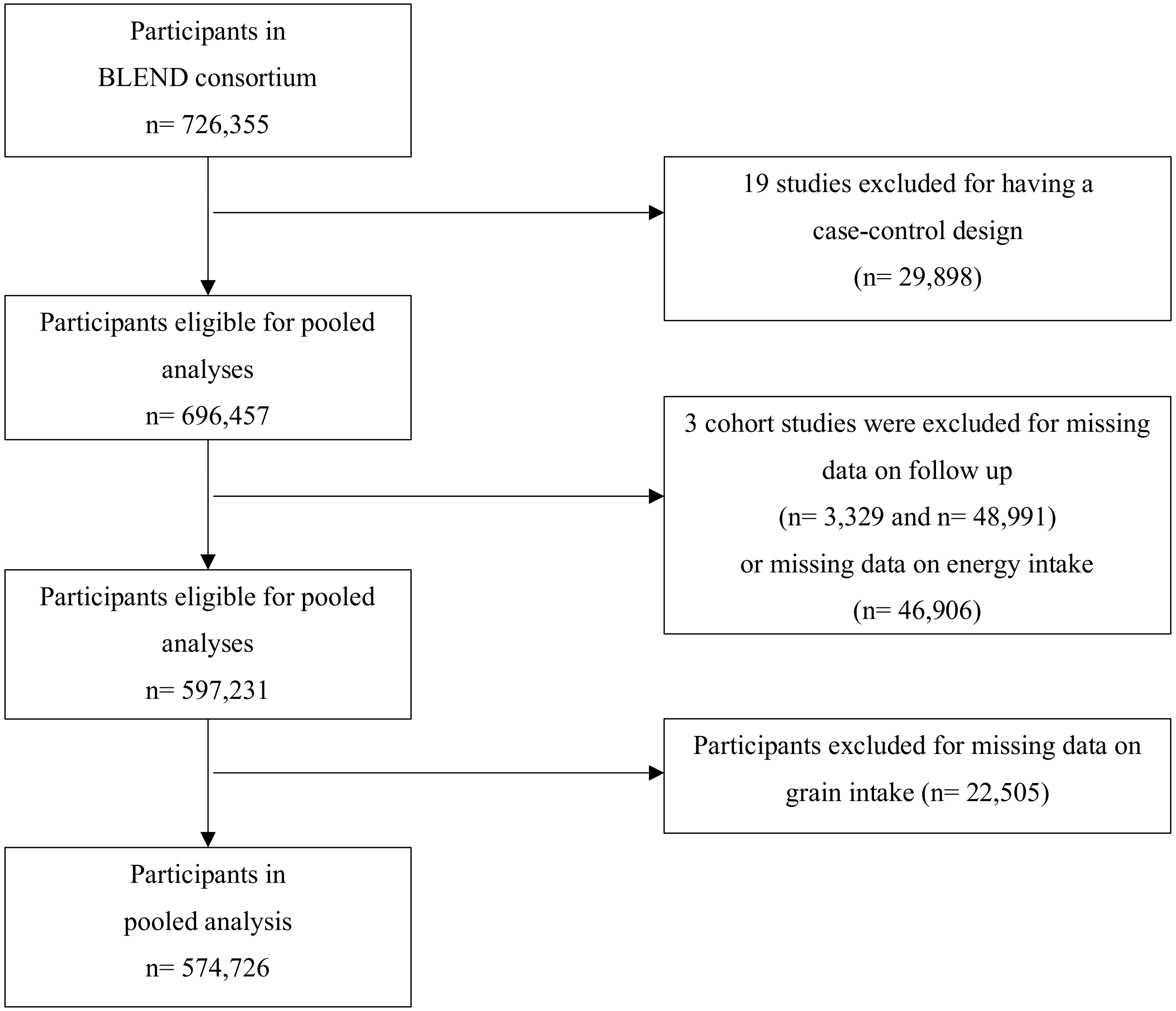

Supplementary Figure 1 Flow diagram of participants included in the statistical analyses on grain and dietary fiber Intake and bladder cancer risk

Abbreviation: BLEND, BLadder cancer Epidemiology and Nutritional Determinants; n, number.

Supplementary Table 1 Additional baseline characteristic of the participant cohort studies

Cohort	No. of Participants (%) [1]	Follow-up Years [2]	Age at Baseline [3]	Person years	No. of Incidences Bladder Cancer (%)	Years to Diagnosis [4]	% of Ever Smokers	Initiate year of baseline assessment
**EPIC (Europe)**								
Denmark	56,005	10.93	50-66	612,129	391	6.82	0.68	1993
France	64,866	10.39	42-71	673,878	31	7.25	0.52	1993
Germany	49,457	9.88	19-70	488,778	207	6.23	0.40	1994
Greece	25,268	9.51	19-84	240,400	50	5.59	0.65	1993
Italy	45,204	11.23	24-78	507,763	187	6.89	0.29	1992
Spain	40,782	12.06	29-70	491,927	152	7.75	0.54	1992
Sweden	49,326	13.12	29-74	647,121	303	8.24	0.44	1991
Netherlands	37,102	11.81	20-70	438,312	107	7.36	0.55	1993
United Kingdom	75,033	11.14	20-98	835,558	248	7.05	0.45	1993
Norway	33,855	9.73	41-56	329,425	24	5.81	0.51	1998
**NLCS (Netherlands)**	5,247	14.06	55-70	73,771	877	9.34	0.62	1986
**VITAL (USA)**	69,491	6.75	50-76	468,779	346	3.87	0.44	2000
**MCCS (Australia)**	23,090	22.86	35-76	527,859	291	14.74	0.64	1990
**Overall**	574,726	11.02	19-99	6,335,667	3,214	8.06	0.50	1986-2000

Abbreviations: EPIC, European Prospective Investigation into Cancer; NLCS, The Netherlands Cohort Study; VITAL, VITamins And Lifestyle study; MCCS, Melbourne Collaborative Cohort Study.

[1] As a result of exclusion criteria, the cohort sizes and number of cases included in the pooled analyses may differ from original study-specific publications.

[2] Median years of the time interval from two years after study entry to the date of the last follow-up (*e.g.* date of death, lost to follow-up, or study exit, whichever came first).

[3] Age ranges at baseline (minimum to maximum).

[4] Time-to-bladder cancer defined by median years of study entry to the date of diagnosis among bladder cancer cases.

Supplementary Table 2 Risk of bladder cancer according to intake of total grain, total whole grain and total refined grain (model 1)

Subgroup		Intake Tertiles	No. Case/ Participants	Model 1 [1]		
				HR (95% CI)	HR Per 1 SD Increase (95% CI)	P-trend
**Total Grains (g/day)**	Overall	Tertile 1	1,005/191,576	Ref.	0.97 (0.92, 1.02)	0.173
		Tertile 2	1,227/191,575	0.93 (0.85, 1.03)		
		Tertile 3	982/191,575	0.95 (0.85, 1.06)		
	MIBC	Tertile 1	263/190,834	Ref.	0.93 (0.82, 1.06)	0.267
		Tertile 2	350/190,698	0.92 (0.76, 1.11)		
		Tertile 3	160/190,753	0.84 (0.66, 1.08)		
	NMIBC	Tertile 1	425/190,996	Ref.	0.99 (0.91, 1.08)	0.810
		Tertile 2	481/190,829	0.97 (0.82, 1.14)		
		Tertile 3	352/190,955	0.95 (0.78, 1.15)		
	Male	Tertile 1	793/62,954	Ref.	0.92 (0.86, 0.99)	0.015
		Tertile 2	1,049/62,954	0.90 (0.80, 1.01)		
		Tertile 3	574/62,954	0.89 (0.78, 1.01)		
	Female	Tertile 1	287/128,622	Ref.	1.08 (0.98, 1.20)	0.118
		Tertile 2	291/128,621	0.98 (0.82, 1.18)		
		Tertile 3	220/128,621	1.12 (0.90, 1.39)		
	Never Smoker	Tertile 1	212/95,457	Ref.	1.01 (0.91, 1.15)	0.804
		Tertile 2	244/95,457	1.04 (0.81, 1.33)		
		Tertile 3	201/95,456	0.95 (0.76, 1.18)		
	Current Smoker	Tertile 1	403/39,716	Ref.	0.99 (0.91, 1.08)	0.776
		Tertile 2	456/39,715	1.06 (0.91, 1.22)		
		Tertile 3	339/39,715	1.03 (0.85, 1.23)		
	Former Smoker	Tertile 1	416/56,404	Ref.	0.91 (0.81, 1.01)	0.423
		Tertile 2	543/56,403	0.85 (0.70, 1.01)		
		Tertile 3	400/56,403	0.85 (0.71, 1.02)		
**Total Whole Grain (g/day)**	Overall	Tertile 1	991/72,821	Ref.	0.94 (0.90, 0.98)	0.013
		Tertile 2	353/74,285	0.97 (0.85, 1.11)		
		Tertile 3	389/70,450	0.86 (0.76, 0.97)		
	MIBC	Tertile 1	360/72,190	Ref.	0.92 (0.85, 0.99)	0.033
		Tertile 2	92/74,024	1.20 (0.95, 1.51)		
		Tertile 3	113/70,174	0.87 (0.70, 1.08)		
	NMIBC	Tertile 1	424/72,254	Ref.	0.96 (0.90, 1.02)	0.210
		Tertile 2	133/72,065	1.07 (0.87, 1.32)		
		Tertile 3	156/70,217	0.84 (0.69, 1.02)		
	Male	Tertile 1	787/22,476	Ref.	0.94 (0.89, 0.99)	0.027

		Tertile 2	259/19,149	0.95 (0.82, 1.10)		
		Tertile 3	295/20,677	0.82 (0.71, 0.95)		
	Female	Tertile 1	204/51,754	Ref.	0.93 (0.86, 1.00)	0.048
		Tertile 2	104/51,830	0.98 (0.84, 1.13)		
		Tertile 3	84/51,670	0.84 (0.73, 0.97)		
	Never Smoker	Tertile 1	188/39,917	Ref.	0.95 (0.86, 1.06)	0.365
		Tertile 2	93/40,808	1.10 (0.85, 1.44)		
		Tertile 3	77/39,024	0.82 (0.62, 1.08)		
	Current Smoker	Tertile 1	362/12,997	Ref.	0.95 (0.89, 1.01)	0.119
		Tertile 2	117/12,699	0.99 (0.79, 1.23)		
		Tertile 3	151/12,836	0.82 (0.62, 1.08)		
	Former Smoker	Tertile 1	425/19,760	Ref.	0.93 (0.87, 1.01)	0.178
		Tertile 2	152/19,970	0.98 (0.81, 1.20)		
		Tertile 3	168/19,545	0.89 (0.74, 1.07)		
**Total Refined Grains (g/day)**	Overall	Tertile 1	1,004/191,576	Ref.	0.98 (0.93, 1.03)	0.334
		Tertile 2	1,238/191,575	0.94 (0.85, 1.03)		
		Tertile 3	972/191,575	0.96 (0.86, 1.08)		
	MIBC	Tertile 1	267/190,839	Ref.	0.97 (0.85, 1.10)	0.612
		Tertile 2	356/190,693	0.93 (0.77, 1.12)		
		Tertile 3	150/190,753	0.86 (0.67, 1.11)		
	NMIBC	Tertile 1	422/190,994	Ref.	1.00 (0.92, 1.09)	0.997
		Tertile 2	491/190,828	1.01 (0.86, 1.18)		
		Tertile 3	355/190,958	0.99 (0.82, 1.21)		
	Male	Tertile 1	808/62,954	Ref.	0.93 (0.87, 1.00)	0.038
		Tertile 2	1,048/62,954	0.90 (0.81, 1.00)		
		Tertile 3	560/62,954	0.89 (0.77, 1.02)		
	Female	Tertile 1	283/128,623	Ref.	1.09 (0.99, 1.21)	0.087
		Tertile 2	295/128,620	1.01 (0.84, 1.21)		
		Tertile 3	220/128,621	1.14 (0.91, 1.48)		
	Never Smoker	Tertile 1	217/95,457	Ref.	1.02 (0.91, 1.14)	0.718
		Tertile 2	232/95,457	0.88 (0.71, 1.08)		
		Tertile 3	208/95,456	1.06 (0.82, 1.35)		
	Current Smoker	Tertile 1	404/39,716	Ref.	1.00 (0.92, 1.09)	0.983
		Tertile 2	464/39,715	1.09 (0.94, 1.26)		
		Tertile 3	330/39,715	1.04 (0.87, 1.25)		
	Former Smoker	Tertile 1	414/56,404	Ref.	0.92 (0.84, 1.00)	0.062
		Tertile 2	552/56,403	0.89 (0.74, 1.07)		

		Tertile 3	393/56,403	0.87 (0.75, 1.02)		

Abbreviation: CI, confidence interval; HR, hazard ratio; kcal, kilocalorie.

The intervals of tertiles were defined as; total grain: 1) overall, 0 ≤ tertile 1 ≤ 105 g/day, 105 < tertile 2 ≤ 186 g/day, tertile 3 > 186 g/day, 2) MIBC, 0 ≤ tertile 1 ≤ 105 g/day, 105 < tertile 2 ≤ 186 g/day, tertile 3 > 186 g/day, 3) NMIBC, 0 ≤ tertile 1 ≤ 105 g/day, 105 < tertile 2 ≤ 186 g/day, tertile 3 > 186 g/day, 4) male, 0 ≤ tertile 1 ≤ 113 g/day, 113 < tertile 2 ≤ 215 g/day, tertile 3 > 215 g/day, 5) female, 0 ≤ tertile 1 ≤ 102 g/day, 102 < tertile 2 ≤ 173 g/day, tertile 3 > 173 g/day, 6) never smoker, 0 ≤ tertile 1 ≤ 104 g/day, 104 < tertile 2 ≤ 181 g/day, tertile 3 > 181 g/day, 7) current smoker, 0 ≤ tertile 1 ≤ 121 g/day, 121 < tertile 2 ≤ 204 g/day, tertile 3 > 204 g/day, 8) former smoker, 0 ≤ tertile 1 ≤ 96 g/day, 96 < tertile 2 ≤ 182 g/day, tertile 3 > 182 g/day; total whole grain: 1) overall, 0 ≤ tertile 1 ≤ 3 g/day, 3 < tertile 2 ≤ 8 g/day, tertile 3 > 8 g/day, 2) MIBC, 0 ≤ tertile 1 ≤ 3 g/day, 3 < tertile 2 ≤ 8 g/day, tertile 3 > 8 g/day, 3) NMIBC, 0 ≤ tertile 1 ≤ 3 g/day, 3 < tertile 2 ≤ 8 g/day, tertile 3 > 8 g/day, 4) male, 0 ≤ tertile 1 ≤ 3 g/day, 3 < tertile 2 ≤ 9 g/day, tertile 3 > 9 g/day, 5) female, 0 ≤ tertile 1 ≤ 3 g/day, 3 < tertile 2 ≤ 8 g/day, tertile 3 > 8 g/day, 6) never smoker, 0 ≤ tertile 1 ≤ 3 g/day, 3 < tertile 2 ≤ 8 g/day, tertile 3 > 8 g/day, 7) current smoker, 0 ≤ tertiel 1 ≤ 3 g/day, 3 < tertile 2 ≤ 8 g/day, tertile 3 > 8 g/day, 8) former smoker, 0 ≤ tertile 1 ≤ 3 g/day, 3 < tertile 2 ≤ 8 g/day, tertile 3 > 8 g/day; total refined grain: 1) overall, 0 ≤ tertile 1 ≤ 102 g/day, 102 < tertile 2 ≤ 181 g/day, tertile 3 > 181 g/day, 2) MIBC, 0 ≤ tertile 1 ≤ 102 g/day, 102 < tertile 2 ≤ 181 g/day, tertile 3 > 181 g/day, 3) NMIBC, 0 ≤ tertile 1 ≤ 102 g/day, 102 < tertile 2 ≤ 181 g/day, tertile 3 > 181 g/day, 4) male, 0 ≤ tertile 1 ≤ 111 g/day, 111 < tertile 2 ≤ 211 g/day, tertile 3 > 211 g/day, 5) female, 0 ≤ tertile 1 ≤ 99 g/day, 99 < tertile 2 ≤ 169 g/day, tertile 3 > 169 g/day, 6) never smoker, 0 ≤ tertile 1 ≤ 100 g/day, 100 < tertile 2 ≤ 176 g/day, tertile 3 > 176 g/day, 7) current smoker, 0 ≤ tertile 1 ≤ 119 g/day, 119 < tertile 2 ≤ 201 g/day, tertile 3 > 201 g/day, 8) former smoker, 0 ≤ tertile 1 ≤ 93 g/day, 93 < tertile 2 ≤ 178 g/day, tertile 3 > 178 g/day.

[1] Model 1 of Cox regression: Adjusted for age (years, continuous), sex (male or female), smoking (smoking was defined as: 0 (never smokers); 1 [current light smokers (*i.e.* smoking less than 20 pack-years)]; 2 [current heavy smokers (*i.e.* smoking more than 20 pack-years)]; 3 [current smokers (no information on pack-years)]; 4 [former light smokers (*i.e.* smokers who ceased smoking over 1 year prior and smoked less than 20 pack-years)]; 5 [former heavy smokers (*i.e.* smokers who ceased smoking over 1 year prior and smoked more than 20 pack-years)]; 6 [former smokers (smokers who ceased smoking over 1 year prior and no information on pack-years)]), and total energy intake (kcal/day, continuous).

Reference group was lowest intake (tertile 1).

P-trend <0.05 was considered statistically significant.

Supplementary Table 3 Risk of bladder cancer according to individual intake of whole grains and refined grains (model 1)

**Subgroup**		**Intake Tertiles**	**No. Case/ Participants**	**Model 1** [1]		
				**HR (95% CI)**	**HR Per 1 SD Increase (95% CI)**	**P-trend**
**Whole Grains (g/day)**	Brown Rice	Tertile 1	910/64,959	Ref.	0.92 (0.86, 0.98)	0.010
		Tertile 2	262/64,685	0.96 (0.82, 1.11)		
		Tertile 3	270/64,822	0.79 (0.68, 0.92)		
	Wheat or Oat	Tertile 1	877/15,715	Ref.	0.98 (0.92, 1.06)	0.644
		Tertile 2	81/3,590	1.21 (0.95, 1.52)		
		Tertile 3	210/9,032	0.92 (0.79, 1.08)		
	Basic Products of Other Cereals [6]	Tertile 1	820/4,802	Ref.	0.98 (0.91, 1.05)	0.555
		Tertile 2	25/233	0.78 (0.52, 1.16)		
		Tertile 3	32/212	1.00 (0.70, 1.42)		
**Refined Grains (g/day)**	White Rice	Tertile 1	976/44,980	Ref.	0.95 (0.88, 1.04)	0.269
		Tertile 2	288/44,951	1.08 (0.92, 1.26)		
		Tertile 3	221/44,954	1.05 (0.91, 1.21)		
	Pasta or Noodles	Tertile 1	806/193,351	Ref.	0.99 (0.94, 1.04)	0.603
		Tertile 2	787/188,377	0.89 (0.81, 0.99)		
		Tertile 3	744/187,751	0.89 (0.79, 1.01)		
	Leavened Bread	Tertile 1	1,057/191,576	Ref.	1.00 (0.95, 1.05)	0.948
		Tertile 2	1,260/191,594	1.01 (0.91, 1.11)		
		Tertile 3	897/191,556	1.02 (0.90, 1.15)		
	Unleavened Bread	Tertile 1	775/119,122	Ref.	0.95 (0.89, 1.00)	0.068
		Tertile 2	939/181,124	0.94 (0.84, 1.05)		
		Tertile 3	863/181,124	0.97 (0.87, 1.09)		
	Bakery Wares	Tertile 1	1,732/477,213	Ref.	0.98 (0.96, 1.01)	0.267
		Tertile 2	688/14,011	1.09 (0.76, 1.55)		
		Tertile 3	448/14,011	1.01 (0.70, 1.45)		
	Savory Cereals Dishes [7]	Tertile 1	161/28,872	Ref.	0.94 (0.82, 1.07)	0.323
		Tertile 2	96/18,996	0.94 (0.73, 1.22)		
		Tertile 3	89/21,623	0.86 (0.66, 1.14)		

	Breakfast Cereals	Tertile 1	1,013/33,151	Ref.	0.96 (0.89, 1.03)	0.280
		Tertile 2	251/32,949	1.00 (0.85, 1.17)		
		Tertile 3	250/31,728	0.94 (0.79, 1.13)		

Abbreviation: CI, confidence interval; HR, hazard ratio; kcal, kilocalorie.

The intervals of tertiles were defined as; total whole grain: 1) brown rice, 0 ≤ tertile 1 ≤ 4 g/day, 4 < tertile 2 ≤ 9 g/day, tertile 3 > 9 g/day, 2) wheat or oat, tertile 1 = 0 g/day, 0 < tertile 2 ≤ 2 g/day, tertile 3 > 2 g/day, 3) basic products of other cereals; total dietary fiber: tertile 1 = 0 g/day, 0 < tertile 2 ≤ 3 g/day, tertile 3 > 3 g/day; total refined grain: 1) white rice, 0 ≤ tertile 1 ≤ 4 g/day, 4 < tertile 2 ≤ 11 g/day, tertile 3 > 11 g/day, 2) pasta or noodles, 0 ≤ tertile 1 ≤ 3 g/day, 3 < tertile 2 ≤ 9 g/day, tertile 3 > 9 g/day, 3) leavened bread, 0 ≤ tertile 1 ≤ 73 g/day, 73 < tertile 2 ≤ 160 g/day, tertile 3 > 160 g/day, 4) unleavened bread, tertile 1 = 0 g/day, 0 < tertile 2 ≤ 4 g/day, tertile 3 > 4 g/day, 5) bakery wares, tertile 1 = 0 g/day, 0 < tertile 2 ≤ 27 g/day, tertile 3 > 27 g/day, 6) savory cereals dishes, 0 ≤ tertile 1 ≤ 3 g/day, 3 < tertile 2 ≤ 7 g/day, tertile 3 > 7 g/day, 7) breakfast cereals, 0 ≤ tertile 1 ≤ 6 g/day, 6 < tertile 2 ≤ 27 g/day, tertile 3 > 27 g/day.

[1] Model 1 of Cox regression: Adjusted for age (years, continuous), sex (male or female), smoking (smoking was defined as: 0 (never smokers); 1 [current light smokers (*i.e.* smoking less than 20 pack-years)]; 2 [current heavy smokers (*i.e.* smoking more than 20 pack-years)]; 3 [current smokers (no information on pack-years)]; 4 [former light smokers (*i.e.* smokers who ceased smoking over 1 year prior and smoked less than 20 pack-years)]; 5 [former heavy smokers (*i.e.* smokers who ceased smoking over 1 year prior and smoked more than 20 pack-years)]; 6 [former smokers (smokers who ceased smoking over 1 year prior and no information on pack-years)]), and total energy intake (kcal/day, continuous).

Reference group was lowest intake (tertile 1).

P-trend <0.05 was considered statistically significant.

Supplementary Table 4 Risk of bladder cancer according to intake of total dietary fiber and individual source of dietary fiber (model 1)

Subgroup		Intake Tertiles	No. Case/ Participants	Model 1 [1]		
				HR (95% CI)	HR Per 1 SD Increase (95% CI	P-trend
**Total Dietary Fiber (g/day)**	Overall	Tertile 1	1,015/191,576	Ref.	0.94 (0.89, 0.99)	0.045
		Tertile 2	1,097/191,575	0.93 (0.84, 1.03)		
		Tertile 3	1,102/191,575	0.87 (0.78, 0.97)		
	Male	Tertile 1	775/62,954	Ref.	0.94 (0.88, 0.99)	0.042
		Tertile 2	971/62,954	0.94 (0.86, 1.03)		
		Tertile 3	670/62,954	0.90 (0.81, 0.99)		
	Female	Tertile 1	322/128,622	Ref.	0.88 (0.77, 0.99)	0.044
		Tertile 2	272/128,621	0.80 (0.66, 0.95)		
		Tertile 3	204/128,621	0.78 (0.63, 0.97)		
	Never Smoker	Tertile 1	242/95,457	Ref.	0.97 (0.85, 1.11)	0.645
		Tertile 2	272/95,457	0.85 (0.68, 1.05)		
		Tertile 3	170/95,456	0.92 (0.73, 1.17)		
	Current Smoker	Tertile 1	436/39,716	Ref.	0.93 (0.85, 1.02)	0.113
		Tertile 2	438/39,715	0.92 (0.78, 1.08)		
		Tertile 3	324/39,715	0.81 (0.68, 0.98)		
	Former Smoker	Tertile 1	473/56,404	Ref.	0.94 (0.86, 1.03)	0.185
		Tertile 2	492/56,403	0.98 (0.84, 1.15)		
		Tertile 3	394/56,403	0.90 (0.75, 1.07)		
**Cereal Fiber (g/day)**	Overall	Tertile 1	1,111/191,576	Ref.	0.97 (0.92, 1.02)	0.195
		Tertile 2	1,203/191,576	0.95 (0.87, 1.04)		
		Tertile 3	900/191,574	0.95 (0.87, 1.04)		
	Male	Tertile 1	869/62,954	Ref.	0.92 (0.86, 1.02)	0.065
		Tertile 2	1,017/62,954	0.96 (0.86, 1.06)		
		Tertile 3	530/62,954	0.91 (0.79, 1.04)		
	Female	Tertile 1	300/128,622	Ref.	0.97 (0.90, 1.07)	0.342
		Tertile 2	293/128,621	1.09 (0.88, 1.37)		
		Tertile 3	205/128,621	1.04 (0.87, 1.24)		
	Never Smoker	Tertile 1	227/95,457	Ref.	1.03 (0.93, 1.14)	0.617
		Tertile 2	245/95,457	1.01 (0.82, 1.24)		
		Tertile 3	185/95,456	1.05 (0.82, 1.36)		
	Current Smoker	Tertile 1	462/39,723	Ref.	0.99 (0.91, 1.08)	0.855
		Tertile 2	416/39,718	1.02 (0.88, 1.18)		
		Tertile 3	320/39,705	1.01 (0.83, 1.20)		
	Former Smoker	Tertile 1	450/56,404	Ref.	0.90 (0.85, 1.02)	0.419
		Tertile 2	557/56,403	0.88 (0.76, 1.02)		
		Tertile 3	352/56,403	0.83 (0.70, 1.01)		

**Fruit Fiber (g/day)**	Overall	Tertile 1	1,059/191,576	Ref.	0.98 (0.95, 1.02)	0.396
		Tertile 2	950/191,613	0.97 (0.88, 1.06)		
		Tertile 3	1,205/191,537	0.96 (0.88, 1.06)		
	Male	Tertile 1	688/62,954	Ref.	1.00 (0.96, 1.04)	0.937
		Tertile 2	689/62,954	0.97 (0.87, 1.09)		
		Tertile 3	1,039/62,954	1.00 (0.90, 1.12)		
	Female	Tertile 1	250/128,628	Ref.	0.93 (0.86, 1.00)	0.051
		Tertile 2	264/128,615	0.94 (0.79, 1.13)		
		Tertile 3	284/128,337	0.78 (0.64, 1.01)		
	Never Smoker	Tertile 1	170/95,459	Ref.	0.97 (0.90, 1.05)	0.446
		Tertile 2	231/95,455	1.16 (0.93, 1.45)		
		Tertile 3	256/95,456	1.07 (0.86, 1.34)		
	Current Smoker	Tertile 1	380/39,716	Ref.	0.98 (0.92, 1.04)	0.460
		Tertile 2	376/39,715	1.02 (0.88, 1.18)		
		Tertile 3	442/39,715	0.90 (0.77, 1.05)		
	Former Smoker	Tertile 1	396/56,413	Ref.	0.99 (0.94, 1.05)	0.819
		Tertile 2	384/56,394	0.92 (0.80, 1.07)		
		Tertile 3	579/56,403	1.00 (0.87, 1.16)		
**Vegetable Fiber (g/day)**	Overall	Tertile 1	1,185/191,576	Ref.	0.99 (0.97-1.03)	0.827
		Tertile 2	1,223/191,575	0.99 (0.90-1.08)		
		Tertile 3	806/191,575	0.92 (0.82-1.02)		
	Male	Tertile 1	718/62,954	Ref.	0.98 (0.92, 1.03)	0.408
		Tertile 2	810/62,954	1.03 (0.92, 1.16)		
		Tertile 3	888/62,954	0.94 (0.82, 1.07)		
	Female	Tertile 1	359/128,622	Ref.	0.87 (0.74, 1.03)	0.122
		Tertile 2	295/128,621	0.88 (0.74, 1.04)		
		Tertile 3	144/128,621	0.76 (0.58, 1.01)		
	Never Smoker	Tertile 1	282/95,457	Ref.	0.98 (0.84, 1.14)	0.757
		Tertile 2	237/95,457	0.95 (0.78, 1.15)		
		Tertile 3	138/95,457	0.98 (0.76, 1.27)		
	Current Smoker	Tertile 1	403/39,716	Ref.	0.89 (0.78, 1.02)	0.239
		Tertile 2	453/39,715	0.94 (0.80, 1.10)		
		Tertile 3	342/39,715	0.82 (0.68, 1.03)		
	Former Smoker	Tertile 1	488/56,404	Ref.	0.98 (0.88, 1.08)	0.631
		Tertile 2	497/56,403	1.03 (0.89, 1.19)		
		Tertile 3	374/56,403	0.94 (0.79, 1.12)		

Abbreviation: CI, confidence interval; HR, hazard ratio; kcal, kilocalorie.

The intervals of tertiles were defined as; total dietary fiber: 1) overall, 0 ≤ tertile 1 ≤ 17 g/day, 17 < tertile 2 ≤ 25 g/day, tertile 3 > 25 g/day, 2) male, 0 ≤ tertile 1 ≤ 17 g/day, 17 < tertile 2 ≤ 26 g/day, tertile 3 > 26 g/day, 3) female, 0 ≤ tertile 1 ≤ 18 g/day, 18 < tertile 2 ≤ 26 g/day, tertile 3 > 26 g/day, 4) never smoker, 0 ≤ tertile 1 ≤ 18 g/day, 18 < tertile 2 ≤ 26 g/day, tertile 3 > 26 g/day, 5) current smoker, 0 ≤ tertile 1 ≤ 17 g/day, 17 < tertile 2 ≤ 25 g/day, tertile 3 > 25 g/day, 6) former smoker, 0 ≤ tertile 1 ≤ 17 g/day, 17 < tertile 2 ≤ 25 g/day, tertile 3 > 25 g/day; cereal fiber: 1) overall, 0 ≤ tertile 1 ≤ 7 g/day, 7 < tertile 2 ≤ 12 g/day, tertile 3 > 12 g/day, 2) male, 0 ≤ tertile 1 ≤ 7 g/day, 7 < tertile 2 ≤ 13 g/day, tertile 3 > 13 g/day, 3) female, 0 ≤ tertile 1 ≤ 6 g/day, 6 < tertile 2 ≤ 11 g/day, tertile 3 > 11 g/day, 4) never smoker, 0 ≤ tertile 1 ≤ 6 g/day, 6 < tertile 2 ≤ 11 g/day, tertile 3 > 11 g/day, 5) current smoker, 0 ≤ tertile 1 ≤ 8 g/day, 8 < tertile 2 ≤ 13 g/day, tertile 3 > 13 g/day, 6) former smoker, 0 ≤ tertile 1 ≤ 6 g/day, 6 < tertile 2 ≤ 11 g/day, tertile 3 > 11 g/day; fruit fiber: 1) overall, 0 ≤ tertile 1 ≤ 2 g/day, 2 < tertile 2 ≤ 4 g/day, tertile 3 > 4 g/day, 2) male, 0 ≤ tertile 1 ≤ 1 g/day, 1 < tertile 2 ≤ 3 g/day, tertile 3 > 3 g/day, 3) female, 0 ≤ tertile 1 ≤ 2 g/day, 2 < tertile 2 ≤ 4 g/day, tertile 3 > 4 g/day, 4) never smoker, 0 ≤ tertile 1 ≤ 2 g/day, 2 < tertile 2 ≤ 4 g/day, tertile 3 > 4 g/day, 5) current smoker, 0 ≤ tertile 1 ≤ 1 g/day, 1 < tertile 2 ≤ 3 g/day, tertile 3 > 3 g/day, 6) former smoker, 0 ≤ tertile 1 ≤ 1 g/day, 1 < tertile 2 ≤ 3 g/day, tertile 3 > 3 g/day; vegetable fiber: 1) overall, 0 ≤ tertile 1 ≤ 5 g/day, 5 < tertile 2 ≤ 9 g/day, tertile 3 > 9 g/day, 2) male, 0 ≤ tertile 1 ≤ 4 g/day, 4 < tertile 2 ≤ 7 g/day, tertile 3 > 7 g/day, 3) female, 0 ≤ tertile 1 ≤ 5 g/day, 5 < tertile 2 ≤ 9 g/day, tertile 3 > 9 g/day, 4) never smoker, 0 ≤ tertile 1 ≤ 5 g/day, 5 < tertile 2 ≤ 9 g/day, tertile 3 > 9 g/day, 5) current smoker, 0 ≤ tertile 1 ≤ 5 g/day, 5 < tertile 2 ≤ 8 g/day, tertile 3 > 8 g/day, 6) former smoker, 0 ≤ tertile 1 ≤ 4 g/day, 4 < tertile 2 ≤ 8 g/day, tertile 3 > 8 g/day.

[1] Model 1 of Cox regression: Adjusted for age (years, continuous), sex (male or female), smoking (smoking was defined as: 0 (never smokers); 1 [current light smokers (*i.e.* smoking less than 20 pack-years)]; 2 [current heavy smokers (*i.e.* smoking more than 20 pack-years)]; 3 [current smokers (no information on pack-years)]; 4 [former light smokers (*i.e.* smokers who ceased smoking over 1 year prior and smoked less than 20 pack-years)]; 5 [former heavy smokers (*i.e.* smokers who ceased smoking over 1 year prior and smoked more than 20 pack-years)]; 6 [former smokers (smokers who ceased smoking over 1 year prior and no information on pack-years)]), and total energy intake (kcal/day, continuous).

Reference group was lowest intake (tertile 1).

P-trend <0.05 was considered statistically significant.

Supplementary Table 5 Joint association of intake of total whole grain and total dietary fiber with bladder cancer risk (model 1)

**Total Dietary Fiber (g/day)**		**Total Whole Grain and Total Dietary Fiber (g/day)**				
		**Model 1** [1]			**P-trend**	**P-interaction**
		Tertile 1	Tertile 2	Tertile 3		
Tertile 1	**No. Case**	348	421	222	0.023	0.015
	**HR (95% CI)**	Ref.	0.92 (0.80, 1.07)	0.88 (0.74, 1.05)		
Tertile 2	**No. Case**	93	157	103		
	**HR (95% CI)**	0.92 (0.73, 1.16)	0.88 (0.73, 1.07)	0.76 (0.60, 0.96)		
Tertile 3	**No. Case**	84	152	153		
	**HR (95% CI)**	0.81 (0.64, 1.03)	0.70 (0.58, 0.85)	0.69 (0.57, 0.86)		

Abbreviation: CI, confidence interval; g, gram; HR, hazard ratio; kcal, kilocalorie; ml, milliliter.

The intervals of tertiles were defined as; total whole grain: 0 ≤ tertil 1 ≤ 3 g/day, 3 < tertil 2 ≤ 8 g/day, tertil 3 > 8 g/day; total dietary fiber: 0 ≤ tertile 1 ≤ 17 g/day, 17 < tertile 2 ≤ 25 g/day, tertile 3 > 25 g/day.

[1] Model 1 of Cox regression: Adjusted for age (years, continuous), sex (male or female), smoking (smoking was defined as: 0 (never smokers); 1 [current light smokers (*i.e.* smoking less than 20 pack-years)]; 2 [current heavy smokers (*i.e.* smoking more than 20 pack-years)]; 3 [current smokers (no information on pack-years)]; 4 [former light smokers (*i.e.* smokers who ceased smoking over 1 year prior and smoked less than 20 pack-years)]; 5 [former heavy smokers (*i.e.* smokers who ceased smoking over 1 year prior and smoked more than 20 pack-years)]; 6 [former smokers (smokers who ceased smoking over 1 year prior and no information on pack-years)]), and total energy intake (kcal/day, continuous).

Reference group was lowest intake (tertile 1).

P-trend <0.05 was considered statistically significant.

Grains and dietary fiber intake and bladder cancer risk: a pooled analysis of prospective cohort studies
Yu et. al
On-line Supplementary Material

Supplementary Table 6 Risk of bladder cancer of each dose-response relationship according to intakes of grain and dietary fiber (model 2)

**Total Whole Grain**		**Total Refined Grain**		**Total Dietary Fiber**		**Cereal Fiber**		**Fruit fiber**		**Vegetable fiber**	
**Dose (g/day)**	**HR (95% CI)**	**Dose (g/day)**	**HR (95% CI)**	**Dose (g/day)**	**HR (95% CI)**	**Dose (g/day)**	**HR (95% CI)**	**Dose (g/day)**	**HR (95% CI)**	**Dose (g/day)**	**HR (95% CI)**
0	Ref.	0	Ref.	0	Ref.	0	Ref.	0	Ref.	0-1	Ref.
0-5	0.99 (0.92, 1.05)	0-30	1.08 (0.94, 1.20)	0-5	0.99 (0.89, 1.07)	0-2	1.04 (0.90, 1.13)	0-1	1.02 (0.87, 1.18)	1-3	0.96 (0.82, 1.08)
5-10	0.95 (0.92, 1.05)	30-60	1.07 (0.95, 1.19)	5-10	0.96 (0.87, 1.05)	2-4	1.06 (0.93, 1.19)	1-2	1.01 (0.84, 1.20)	3-5	0.93 (0.77, 1.04)
10-15	0.91 (0.88, 1.02)	60-90	1.04 (0.93, 1.16)	10-15	0.93 (0.84, 1.03)	4-6	1.08 (0.95, 1.20)	2-3	1.00 (0.82, 1.21)	5-7	0.90 (0.74, 1.03)
15-20	0.88 (0.83, 0.99)	90-120	1.02 (0.90, 1.14)	15-20	0.91 (0.79, 1.01)	6-8	1.10 (0.97, 1.21)	3-4	0.99 (0.79, 1.20)	7-9	0.87 (0.71, 1.02)
15-25	0.84 (0.75, 0.93)	120-150	0.99 (0.87, 1.11)	15-25	0.87 (0.75, 1.00)	8-10	1.11 (0.99, 1.23)	4-5	0.99 (0.77, 1.20)	9-11	0.85 (0.70, 1.01)
25-30	0.81 (0.70, 0.92)	150-180	0.97 (0.85, 1.09)	25-30	0.83 (0.71, 0.97)	10-12	1.10 (0.95, 1.23)	5-6	0.98 (0.74, 1.20)	11-13	0.85 (0.68, 1.01)
30-35	0.78 (0.66, 0.90)	180-210	0.95 (0.83, 1.07)	30-35	0.80 (0.67, 0.94)	12-14	1.07 (0.91, 1.21)	6-7	0.98 (0.72, 1.21)	13-15	0.84 (0.65, 1.02)
35-40	0.76 (0.62, 0.90)	210-240	0.93 (0.81, 1.05)	35-40	0.77 (0.61, 0.92)	14-16	1.01 (0.84, 1.18)	7-8	0.98 (0.73, 1.21)	15-17	0.84 (0.62, 1.02)
40-45	0.73 (0.57, 0.89)	240-270	0.91 (0.79, 1.04)	40-45	0.74 (0.55, 0.91)	16-18	0.93 (0.74, 1.13)	8-9	0.99 (0.71, 1.23)	17-19	0.83 (0.59, 1.03)
45-50	0.71 (0.53, 0.89)	270-300	0.90 (0.76, 1.04)	45-50	0.72 (0.46, 0.92)	18-20	0.82 (0.59, 1.05)	9-10	1.02 (0.74, 1.21)	19-21	0.81 (0.55, 1.04)

Abbreviation: CI, confidence interval; g, gram; HR, hazard ratio; kcal, kilocalorie; ml, milliliter.

[1] Model 2 of Cox regression: Adjusted for age (years, continuous), sex (male or female), smoking (smoking was defined as: 0 (never smokers); 1 [current light smokers (*i.e.* smoking less than 20 pack-years)]; 2 [current heavy smokers (*i.e.* smoking more than 20 pack-years)]; 3 [current smokers (no information on pack-years)]; 4 [former light smokers (*i.e.* smokers who ceased smoking over 1 year prior and smoked less than 20 pack-years)]; 5 [former heavy smokers (*i.e.* smokers who ceased smoking over 1 year prior and smoked more than 20 pack-years)]; 6 [former smokers (smokers who ceased smoking over 1 year prior and no information on pack-years)]), total energy intake (kcal/day, continuous), ethnicity (Caucasian or non-Caucasian, if applicable) , alcohol intake (ml/day, continuous), fruit intake (g/day, continuous), fat intake (g/day, continuous), meat intake (g/day, continuous), sugar intake (g/day, continuous), vegetable intake (g/day, continuous) and total fluid intake (ml/day, continuous).

The intervals of tertiles were categorized as open lower values and ended upper values.

Reference group was non-intake.

Grains and dietary fiber intake and bladder cancer risk: a pooled analysis of prospective cohort studies
Yu et. al
On-line Supplementary Material

(A) Fruit Fiber
Non-linear Test: *P*-test, 0.378
1 g/day Increment Risk: $HR_{-increment}$: 1.00, 95% CI: 0.97, 1.03
P-trend: 0.873

(B) Vegetable Fiber
Non-linear Test: *P*-test: 0.265
2 g/day Increment Risk: $HR_{-increment}$: 0.98, 95% CI: 0.94, 1.05
P-trend: 0.119

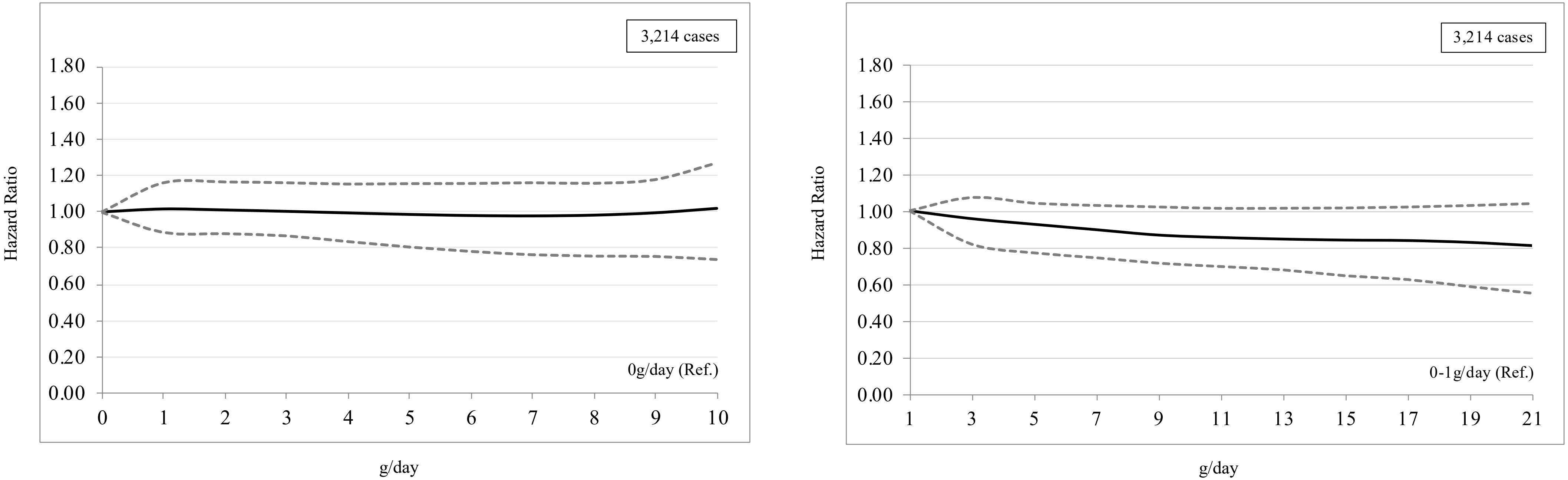

Supplementary Figure 2 Dose-response relationships between dietary fiber intake and the risk of bladder cancer among (A) fruit fiber and (B) vegetable fiber

Abbreviation: CI, confidence interval; g, gram; HR, hazard ratio; kcal, kilocalorie; ml, milliliter.

The solid lines represent the Hazard Ratios (HRs); the dashed lines represent the 95% confidence intervals (CIs) for the trend.

The HRs were adjusted for age (years, continuous), sex (male or female), smoking (smoking was defined as: 0 (never smokers); 1 [current light smokers (*i.e.* smoking less than 20 pack-years)]; 2 [current heavy smokers (*i.e.* smoking more than 20 pack-years)]; 3 [current smokers (no information on pack-years)]; 4 [former light smokers (*i.e.* smokers who ceased smoking over 1 year and smoked less than 20 pack-years)]; 5 [former heavy smokers (*i.e.* smokers who ceased smoking over 1 year and smoked more than 20 pack-years)]; 6 [former smokers (smokers who ceased smoking over 1 year, and no information on pack-years)]), ethnicity (Caucasian or non-Caucasian), total energy intake (kcal/day, continuous), alcohol intake (ml/day, continuous), sugar intake (g/day, continuous), meat intake (g/day, continuous), vegetable intake (g/day, continuous), fruit intake (g/day, continuous), fat intake (g/day, continuous) and total fluid intake (ml/day, continuous) (model 2).

P-test <0.05 were considered statistically significant for non-linearity.

P-increment <0.05 were considered statistically significant.

Supplementary Table 7 Risk of bladder cancer according to grains and dietary fiber intake (after removing 358 cases diagnosed within 2 years)

Subgroup	Intake Tertiles	No. Case/ Participants	Model 1 [1]			Model 2 [1, 2]		
			HR (95% CI)	HR Per 1 SD Increase (95% CI)	P-trend	HR (95% CI)	HR Per 1 SD Increase (95% CI)	P-trend
**Total Whole Grains (g/day)**	Tertile 1	910/72,740	Ref.	0.95 (0.90, 0.99)	0.024	Ref.	0.95 (0.91, 1.00)	0.040
	Tertile 2	323/74,255	0.98 (0.85, 1.12)			0.97 (0.76, 1.10)		
	Tertile 3	360/70,421	0.83 (0.73, 0.94)			0.81 (0.67, 0.97)		
**Total Refined Grains (g/day)**	Tertile 1	863/191,435	Ref.	0.99 (0.94, 1.04)	0.701	Ref.	0.98 (0.93, 1.04)	0.591
	Tertile 2	1,117/191,454	0.94 (0.85, 1.04)			0.93 (0.84, 1.03)		
	Tertile 3	876/191,479	0.97 (0.86, 1.10)			0.96 (0.85, 1.09)		
**Total Dietary Fiber (g/day)**	Tertile 1	1,015/191,417	Ref.	0.93 (0.87, 0.99)	0.020	Ref.	0.86 (0.78, 0.94)	0.002
	Tertile 2	1,091/191,462	0.93 (0.84, 1.04)			0.92 (0.83, 1.03)		
	Tertile 3	750/191,489	0.89 (0.80, 0.97)			0.85 (0.76, 0.96)		
**Cereal Fiber (g/day)**	Tertile 1	967/191,432	Ref.	0.98 (0.93, 1.04)	0.508	Ref.	0.98 (0.92, 1.03)	0.397
	Tertile 2	1,078/191,451	0.98 (0.87, 1.11)			0.97 (0.85, 1.10)		
	Tertile 3	811/191,485	0.94 (0.85, 1.03)			0.94 (0.85, 1.03)		
**Fruit Fiber (g/day)**	Tertile 1	912/191,429	Ref.	0.98 (0.94, 1.02)	0.298	Ref.	0.98 (0.89, 1.07)	0.603
	Tertile 2	848/191,511	0.99 (0.90, 1.09)			1.04 (0.91, 1.18)		
	Tertile 3	1,096/191,428	0.99 (0.89, 1.09)			0.99 (0.91, 1.11)		
**Vegetable Fiber (g/day)**	Tertile 1	1,058/191,449	Ref.	0.92 (0.85, 1.00)	0.059	Ref.	0.91 (0.85, 1.00)	0.048
	Tertile 2	1,074/191,426	0.97 (0.88, 1.07)			0.97 (0.88, 1.08)		
	Tertile 3	724/191,493	0.92 (0.82, 1.04)			0.93 (0.80, 1.07)		

Abbreviation: CI, confidence interval; g, gram; HR, hazard ratio; kcal, kilocalorie; ml, milliliter.

The intervals of tertiles were defined as; total whole grain: 0 ≤ tertile 1 ≤ 3 g/day, 3 < tertile 2 ≤ 8 g/day, tertile 3 > 8 g/day; total refined grain: 0 ≤ tertile 1 ≤ 102 g/day, 102 < tertile 2 ≤ 181 g/day, tertile 3 > 181 g/day; total dietary fiber: 0 ≤ tertile 1 ≤ 17 g/day, 17 < tertile 2 ≤ 25 g/day, tertile 3 > 25 g/day; cereal fiber: 0 ≤ tertile 1 ≤ 7 g/day, 7 < tertile 2 ≤ 12 g/day, tertile 3 > 12 g/day; fruit fiber: 0 ≤ tertile 1 ≤ 2 g/day, 2 < tertile 2 ≤ 4 g/day, tertile 3 > 4 g/day; vegetable fiber: 0 ≤ tertile 1 ≤ 5 g/day, 5 < tertile 2 ≤ 9 g/day, tertile 3 > 9 g/day.

[1] Model 1: Adjusted for age (years, continuous), sex (male or female), smoking (smoking was defined as: 0 (never smokers); 1 [current light smokers (*i.e.* smoking less than 20 pack-years)]; 2 [current heavy smokers (*i.e.* smoking more than 20 pack-years)]; 3 [current smokers (no information on pack-years)]; 4 [former light smokers (*i.e.* smokers who ceased smoking over 1 year prior and smoked less than 20 pack-years)]; 5 [former heavy smokers (*i.e.* smokers who ceased smoking over 1 year prior and smoked more than 20 pack-years)]; 6 [former smokers (smokers who ceased smoking over 1 year prior and no information on pack-years)]), and total energy intake (kcal/day, continuous).

[1, 2] Model 2: Additionally, ethnicity (Caucasian or non-Caucasian, if applicable) , alcohol intake (ml/day, continuous), fruit intake (g/day, continuous), fat intake (g/day, continuous), meat intake (g/day, continuous), sugar intake (g/day, continuous), vegetable intake (g/day, continuous) and total fluid intake (ml/day, continuous).

Reference group was lowest intake (tertile 1).

P-trend <0.05 was considered statistically significant.

Supplementary Table 8 Risk of bladder cancer according to intakes of grain and dietary fiber based on complete dataset (model 2)

Overall	Case/Total	Tertile 1	Tertile 2	Tertile 3	HR Per 1 SD Increase (95% CI)	*P*-trend
**Total Grains (g/day)**	2,506/418,897	Ref.	0.89 (0.80, 1.01)	0.91 (0.80, 1.05)	0.97 (0.91, 1.03)	0.314
**Total Whole Drain (g/day)**	1,243/154,280	Ref.	1.06 (0.91, 1.23)	0.87 (0.75, 0.99)	0.94 (0.89, 0.99)	0.024
**Total Refined Drain (g/day)**	2,506/418,897	Ref.	0.90 (0.80, 1.01)	0.92 (0.80, 1.06)	0.98 (0.92, 1.04)	0.521
**Total Dietary Fiber (g/day)**	2,506/418,897	Ref.	0.91 (0.81, 1.03)	0.84 (0.72, 0.98)	0.88 (0.79, 1.00)	0.29
**Cereal Fiber (g/day)**	2,506/418,897	Ref.	0.95 (0.85, 1.06)	0.98 (0.86, 1.12)	0.98 (0.92, 1.04)	0.501
**Fruit Fiber (g/day)**	2,506/418,897	Ref.	0.96 (0.86, 1.07)	0.93 (0.80, 1.09)	0.99 (0.87, 1.12)	0.839
**Vegetable Fiber (g/day)**	2,506/418,897	Ref.	0.97 (0.87, 1.08)	0.94 (0.81, 1.10)	0.91 (0.82, 1.00)	0.041

Abbreviation: CI, confidence interval; g, gram; HR, hazard ratio; kcal, kilocalorie; ml, milliliter.

The intervals of tertiles were defined as; total whole grain: 0 ≤ tertile 1 ≤ 3 g/day, 3 < tertile 2 ≤ 8 g/day, tertile 3 > 8 g/day; total refined grain: 0 ≤ tertile 1 ≤ 102 g/day, 102 < tertile 2 ≤ 181 g/day, tertile 3 > 181 g/day; total dietary fiber: 0 ≤ tertile 1 ≤ 17 g/day, 17 < tertile 2 ≤ 25 g/day, tertile 3 > 25 g/day; cereal fiber: 0 ≤ tertile 1 ≤ 7 g/day, 7 < tertile 2 ≤ 12 g/day, tertile 3 > 12 g/day; fruit fiber: 0 ≤ tertile 1 ≤ 2 g/day, 2 < tertile 2 ≤ 4 g/day, tertile 3 > 4 g/day; vegetable fiber: 0 ≤ tertile 1 ≤ 5 g/day, 5 < tertile 2 ≤ 9 g/day, tertile 3 > 9 g/day.

[1, 2] Model 2 of Cox regression: Adjusted for age (years, continuous), sex (male or female), smoking (smoking was defined as: 0 (never smokers); 1 [current light smokers (*i.e.* smoking less than 20 pack-years)]; 2 [current heavy smokers (*i.e.* smoking more than 20 pack-years)]; 3 [current smokers (no information on pack-years)]; 4 [former light smokers (*i.e.* smokers who ceased smoking over 1 year prior and smoked less than 20 pack-years)]; 5 [former heavy smokers (*i.e.* smokers who ceased smoking over 1 year prior and smoked more than 20 pack-years)]; 6 [former smokers (smokers who ceased smoking over 1 year prior and no information on pack-years)]), total energy intake (kcal/day, continuous), ethnicity (Caucasian or non-Caucasian, if applicable) , alcohol intake (ml/day, continuous), fruit intake (g/day, continuous), fat intake (g/day, continuous), meat intake (g/day, continuous), sugar intake (g/day, continuous), vegetable intake (g/day, continuous) and total fluid intake (ml/day, continuous).

Reference group was non-intake.

Supplementary Table 9 Risk of bladder cancer according to intakes of grain and dietary fiber based on quintile analyses

**Intake Quintiles**		**No. Case/ Participants**	**Model 1 [5]**		**Model 2 [5, 6]**	
			**HR (95% CI)**	**P-trend**	**HR (95% CI)**	**P-trend**
**Total Whole Grains (g/day)**	Quintile 1	875/60,397	Ref.	0.021	Ref.	0.032
	Quintile 2	296/48,419	0.97 (0.85, 1.12)		0.98 (0.85, 1.12)	
	Quintile 3	266/54,459	0.93 (0.80, 1.08)		0.93 (0.81, 1.08)	
	Quintile 4	296/54,281	0.85 (0.74, 0.97)		0.86 (0.75, 0.98)	
**Total Refined Grains (g/day)**	Quintile 1	687/143,682	Ref.	0.307	Ref.	0.215
	Quintile 2	978/143,681	0.93 (0.83, 1.05)		0.93 (0.83, 1.04)	
	Quintile 3	868/143,682	0.91 (0.80, 1.03)		0.90 (0.79, 1.02)	
	Quintile 4	681/143,681	0.93 (0.81, 1.07)		0.91 (0.79, 1.06)	
**Total Dietary Fiber (g/day)**	Quintile 1	878/143,682	Ref.	0.024	Ref.	0.022
	Quintile 2	906/143,681	0.92 (0.84, 1.02)		0.92 (0.83, 1.02)	
	Quintile 3	862/143,682	0.90 (0.81, 1.00)		0.88 (0.78, 0.99)	
	Quintile 4	568/143,681	0.86 (0.76, 0.98)		0.84 (0.72, 0.98)	
**Cereal Fiber (g/day)**	Quintile 1	740/143,682	Ref.	0.290	Ref.	0.196
	Quintile 2	1,000/143,681	0.96 (0.86, 1.07)		0.96 (0.86, 1.07)	
	Quintile 3	822/143,682	0.93 (0.82, 1.04)		0.92 (0.81, 1.03)	
	Quintile 4	652/143,681	0.94 (0.82, 1.08)		0.92 (0.80, 1.06)	
**Fruit Fiber (g/day)**	Quintile 1	792/143,682	Ref.	0.214	Ref.	0.321
	Quintile 2	767/143,681	1.02 (0.92, 1.13)		1.02 (0.92, 1.13)	
	Quintile 3	729/143,682	0.95 (0.85, 1.06)		0.95 (0.85, 1.06)	
	Quintile 4	926/143,681	0.95 (0.85, 1.06)		0.94 (0.81, 1.09)	
**Vegetable Fiber (g/day)**	Quintile 1	932/143,682	Ref.	0.101	Ref.	0.105
	Quintile 2	857/143,681	0.96 (0.87, 1.07)		0.96 (0.86, 1.06)	
	Quintile 3	918/143,682	0.92 (0.83, 1.04)		0.92 (0.81, 1.03)	
	Quintile 4	507/143,681	0.90 (0.79, 1.03)		0.88 (0.75, 1.04)	

Abbreviation: CI, confidence interval; g, gram; HR, hazard ratio; kcal, kilocalorie; ml, milliliter.

The intervals of quintiles were defined as; total whole grain: 0 ≤ quintile 1 ≤ 2 g/day, 2 < quintile 2 ≤ 6 g/day, 6 < quintile 3 ≤ 12 g/day, quintile 4 > 12 g/day; total refined grain: 0 ≤ quintile 1 ≤ 81 g/day, 81 < quintile 2 ≤ 138 g/day, 138 < quintile 3 ≤ 212 g/day, quintile 4 > 212 g/day; total dietary fiber: 0 ≤ quintile 1 ≤ 13 g/day, 13 < quintile 2 ≤ 17 g/day, 13 < quintile 3 ≤ 19 g/day, quintile 4 > 25 g/day; cereal fiber: 0 ≤ quintile 1 ≤ 5 g/day, 5 < quintile 2 ≤ 9 g/day, 9 < quintile 3 ≤ 13 g/day, quintile 4 > 13 g/day; fruit fiber: 0 ≤ quintile 1 ≤ 1 g/day, 1 < quintile 2 ≤ 2 g/day, 2 < quintile 3 ≤ 4 g/day, quintile 4 > 4 g/day; vegetable fiber: 0 ≤ quintile 1 ≤ 4 g/day, 4 < quintile 2 ≤ 6 g/day, 6 < quintile 3 ≤ 10 g/day, quintile 4 > 10 g/day.

[1] Model 1 of Cox regression: Adjusted for age (years, continuous), sex (male or female), smoking (smoking was defined as: 0 (never smokers); 1 [current light smokers (*i.e.* smoking less than 20 pack-years)]; 2 [current heavy smokers (*i.e.* smoking more than 20 pack-years)]; 3 [current smokers (no information on pack-years)]; 4 [former light smokers (*i.e.* smokers who ceased smoking

over 1 year prior and smoked less than 20 pack-years)]; 5 [former heavy smokers (*i.e.* smokers who ceased smoking over 1 year prior and smoked more than 20 pack-years)]; 6 [former smokers (smokers who ceased smoking over 1 year prior and no information on pack-years)]), and total energy intake (kcal/day, continuous).

[1, 2] Model 2: Additionally, ethnicity (Caucasian or non-Caucasian, if applicable) , alcohol intake (ml/day, continuous), fruit intake (g/day, continuous), fat intake (g/day, continuous), meat intake (g/day, continuous), sugar intake (g/day, continuous), vegetable intake (g/day, continuous) and total fluid intake (ml/day, continuous).

Reference group was lowest intake (quintile 1).

P-trend <0.05 was considered statistically significant.

Supplementary Table 10 Risk of bladder cancer risk according to intake of total refined grain (after removing pasta intake)

Subgroup	Intake Tertiles	No. Case/ Participants	Model 1 [1]			Model 2 [1, 2]		
			HR (95% CI)	HR Per 1 SD Increase (95% CI)	P-trend	HR (95% CI)	HR Per 1 SD Increase (95% CI)	P-trend
**Overall**	Tertile 1	917/191,576	Ref.	0.98 (0.93, 1.03)	0.365	Ref.	0.98 (0.92, 1.03)	0.405
	Tertile 2	1,284/191,575	0.98 (0.89, 1.08)			0.96 (0.86, 1.08)		
	Tertile 3	1,013/191,575	1.00 (0.89, 1.12)			0.97 (0.85, 1.11)		
**MIBC**	Tertile 1	233/190,892	Ref.	0.93 (0.81, 1.06)	0.273	Ref.	0.92 (0.78, 1.07)	0.348
	Tertile 2	379/190,670	0.99 (0.81, 1.21)			0.96 (0.76, 1.22)		
	Tertile 3	161/190,723	0.85 (0.66, 1.11)			0.77 (0.57, 1.05)		
**NMIBC**	Tertile 1	395/191,054	Ref.	0.96 (0.88, 1.04)	0.335	Ref.	0.94 (0.86, 1.04)	0.234
	Tertile 2	503/190,794	0.97 (0.82, 1.15)			0.97 (0.79, 1.18)		
	Tertile 3	370/190,932	0.92 (0.76, 1.13)			0.87 (0.69, 1.10)		
**Male**	Tertile 1	712/62,964	Ref.	0.94 (0.89, 1.00)	0.050	Ref.	0.94 (0.89, 1.01)	0.072
	Tertile 2	1,112/62,944	0.92 (0.82, 1.03)			0.92 (0.81, 1.04)		
	Tertile 3	592/62,954	0.91 (0.80, 1.05)			0.91 (0.78, 1.07)		
**Female**	Tertile 1	271/128,622	Ref.	1.06 (0.94, 1.18)	0.354	Ref.	1.06 (0.93, 1.20)	0.380
	Tertile 2	304/128,621	0.99 (0.82, 1.19)			0.93 (0.76, 1.14)		
	Tertile 3	223/128,621	1.10 (0.88, 1.38)			1.11 (0.87, 1.41)		
**Never Smoker**	Tertile 1	206/95,463	Ref.	1.03 (0.92, 1.15)	0.630	Ref.	1.02 (0.91, 1.14)	0.725
	Tertile 2	233/95,451	0.87 (0.70, 1.09)			0.86 (0.69, 1.08)		
	Tertile 3	218/95,456	1.11 (0.86, 1.42)			1.09 (0.84, 1.41)		
**Current Smoker**	Tertile 1	712/62,964	Ref.	0.94 (0.89, 1.00)	0.050	Ref.	0.94 (0.89, 1.01)	0.072
	Tertile 2	379/39,716	1.06 (0.91, 1.23)			0.92 (0.81, 1.04)		
	Tertile 3	480/39,715	0.99 (0.83, 1.20)			0.91 (0.78, 1.07)		
**Former Smoker**	Tertile 1	339/39,715	Ref.	1.00 (0.92, 1.08)	0.938	Ref.	0.98 (0.90, 1.06)	0.599
	Tertile 2	304/128,621	0.99 (0.82, 1.19)			1.04 (0.90, 1.21)		
	Tertile 3	223/128,621	1.10 (0.88, 1.38)			0.96 (0.80, 1.16)		

Abbreviation: CI, confidence interval; g, gram; HR, hazard ratio; kcal, kilocalorie; ml, milliliter.

The intervals of tertiles were defined as; total refined grain: 1) overall, 0 ≤ tertile 1 ≤ 92 g/day, 92 < tertile 2 ≤ 172 g/day, tertile 3 > 172 g/day, 2) MIBC, 0 ≤ tertile 1 ≤ 92 g/day, 92 < tertile 2 ≤ 172 g/day, tertile 3 > 172 g/day, 3) NMIBC, 0 ≤ tertile 1 ≤ 92 g/day, 92 < tertile 2 ≤ 172 g/day, tertile 3 > 172 g/day, 4) male, 0 ≤ tertile 1 ≤ 98 g/day, 98 < tertile 2 ≤ 199 g/day, tertile 3 > 199 g/day, 5) female, 0 ≤ tertile 1 ≤ 90 g/day, 90 < tertile 2 ≤ 159 g/day, tertile 3 > 159 g/day, 6) never smoker, 0 ≤ tertile 1 ≤ 91 g/day, 91 < tertile 2 ≤ 167 g/day, tertile 3 > 167 g/day, 7) current smoker, 0 ≤ tertile 1 ≤ 111 g/day, 111 < tertile 2 ≤ 189 g/day, tertile 3 > 189 g/day, 8) former smoker, 0 ≤ tertile 1 ≤ 92 g/day, 92 < tertile 2 ≤ 172 g/day, tertile 3 > 172 g/day.

[1] Model 1 of Cox regression: Adjusted for age (years, continuous), sex (male or female, if applicable), smoking (smoking was defined as: 0 (never smokers); 1 [current light smokers (*i.e.* smoking less than 20 pack-years)]; 2 [current heavy smokers (*i.e.* smoking more than 20 pack-years)]; 3 [current smokers (no information on pack-years)]; 4 [former light smokers (*i.e.* smokers who ceased

smoking over 1 year prior and smoked less than 20 pack-years)]; 5 [former heavy smokers (*i.e.* smokers who ceased smoking over 1 year prior and smoked more than 20 pack-years)]; 6 [former smokers (smokers who ceased smoking over 1 year prior and no information on pack-years)]), and total energy intake (kcal/day, continuous).

[1, 2] Model 2: Additionally, ethnicity (Caucasian or non-Caucasian, if applicable) , alcohol intake (ml/day, continuous), fruit intake (g/day, continuous), fat intake (g/day, continuous), meat intake (g/day, continuous), sugar intake (g/day, continuous), vegetable intake (g/day, continuous) and total fluid intake (ml/day, continuous).

Reference group was lowest intake (tertile 1).

P-trend <0.05 was considered statistically significant.

Supplementary Table 11 Risk of bladder cancer according to intake of total whole grain, total refined grain and total dietary fiber in different adjustment models

**Adjustment Models**		**Total Whole Grain (g/day)**			**Total Refined Grain (g/day)**				**Total Dietary Fiber (g/day)**			
	Case/Total	Tertile 1	Tertile 2	Tertile 3	Case/Total	Tertile 1	Tertile 2	Tertile 3	Case/Total	Tertile 1	Tertile 2	Tertile 3
Model 1) [1]	1,733/217,556	Ref.	0.98 (0.86, 1.11)	0.83 (0.73, 0.94)	3,214/574,726	Ref.	0.93 (0.84, 1.02)	0.94 (0.84, 1.05)	3,214/574,726	Ref.	0.92 (0.83, 1.01)	0.86 (0.77, 0.96)
Model 2) [2]	1,211/146,385	Ref.	0.99 (0.87, 1.12)	0.84 (0.74, 0.95)	2,228/418,548	Ref.	0.93 (0.85, 1.03)	0.95 (0.84, 1.06)	2,228/418,548	Ref.	0.93 (0.84, 1.03)	0.87 (0.78, 0.98)
Model 3) [3]	1,211/146,385	Ref.	0.97 (0.85, 1.10)	0.81 (0.72, 0.92)	2,228/418,548	Ref.	0.93 (0.85, 1.03)	0.95 (0.85, 1.06)	2,228/418,548	Ref.	0.93 (0.84, 1.03)	0.88 (0.79, 0.97)

Abbreviation: CI, confidence interval; g, gram; HR, hazard ratio; kcal, kilocalorie; ml, milliliter.

The intervals of tertiles were defined as; total whole grain: 0 ≤ tertile 1 ≤ 3 g/day, 3 < tertile 2 ≤ 8 g/day, tertile 3 > 8 g/day; total refined grain: 0 ≤ tertile 1 ≤ 102 g/day, 102 < tertile 2 ≤ 181 g/day, tertile 3 > 181 g/day; total dietary fiber: 0 ≤ tertile 1 ≤ 17 g/day, 17 < tertile 2 ≤ 25 g/day, tertile 3 > 25 g/day.

Adjustments of Cox regression: age (years, continuous), sex (male or female), smoking (if applicable, smoking was defined as: 0 (never smokers); 1 [current light smokers (*i.e.* smoking less than 20 pack-years)]; 2 [current heavy smokers (*i.e.* smoking more than 20 pack-years)]; 3 [current smokers (no information on pack-years)]; 4 [former light smokers (*i.e.* smokers who ceased smoking over 1 year prior and smoked less than 20 pack-years)]; 5 [former heavy smokers (*i.e.* smokers who ceased smoking over 1 year prior and smoked more than 20 pack-years)]; 6 [former smokers (smokers who ceased smoking over 1 year prior and no information on pack-years)]), total energy intake (kcal/day, continuous), smoking status (never, former, current), and smoking pack-years (continuous).

[1] Model 1) of Cox regression: adjusted for age, sex energy intake and smoking status.

[2] Model 2) of Cox regression: adjusted for age, sex, energy intake and smoking pack-years.

[3] Model 3) of Cox regression: adjusted for age, sex, energy intake, smoking status and smoking pack-years.

The intervals of tertiles were categorized as open lower values and ended upper values.

Reference group was lowest intake (tertile 1).

P-trend <0.05 was considered statistically significant.

Grains and dietary fiber intake and bladder cancer risk: a pooled analysis of prospective cohort studies
Yu et. al
On-line Supplementary Material

(A) Total Whole Grain

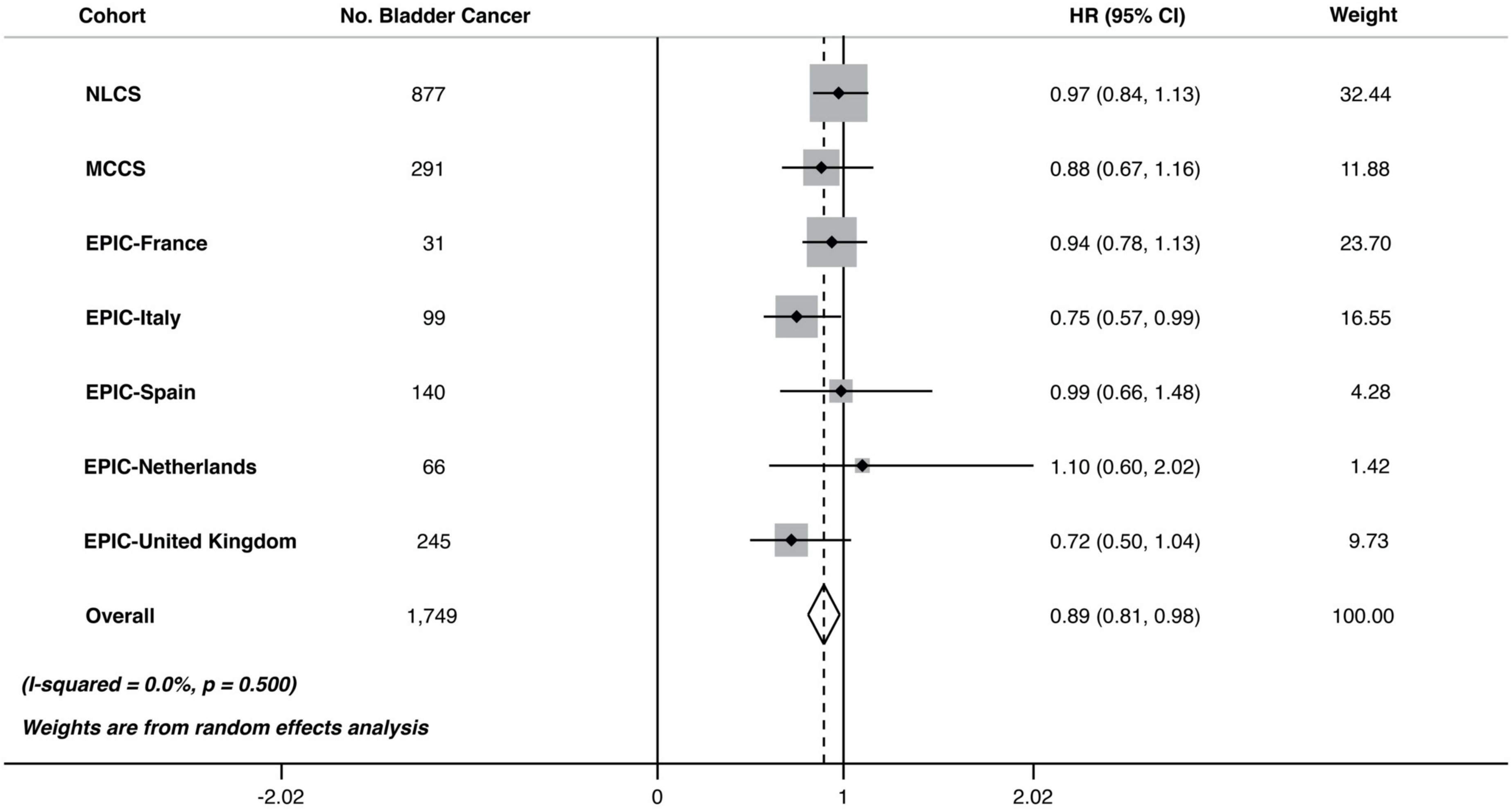

Grains and dietary fiber intake and bladder cancer risk: a pooled analysis of prospective cohort studies
Yu et. al
On-line Supplementary Material

(B) Total Refined Grain

Cohort	No. Bladder Cancer	HR (95% CI)	Weight
NLCS	877	0.80 (0.66, 0.96)	20.25
VITAL	346	1.01 (0.75, 1.35)	10.41
MCCS	291	1.10 (0.82, 1.48)	9.11
EPIC-Denmark	391	1.08 (0.82, 1.43)	9.98
EPIC-France	31	0.87 (0.33, 2.30)	1.41
EPIC-Germany	207	1.26 (0.88, 1.80)	5.46
EPIC-Greece	50	0.85 (0.40, 1.79)	2.69
EPIC-Italy	187	2.23 (1.37, 3.63)	1.09
EPIC-Spain	152	0.62 (0.39, 0.99)	10.43
EPIC-Sweden	303	0.88 (0.63, 1.22)	10.55
EPIC-Netherlands	107	0.71 (0.40, 1.26)	6.16
EPIC-UK	248	0.83 (0.60, 1.15)	11.60
EPIC-Norway	24	0.91 (0.29, 2.83)	0.86
Overall	3,214	0.91 (0.79, 1.03)	100.00

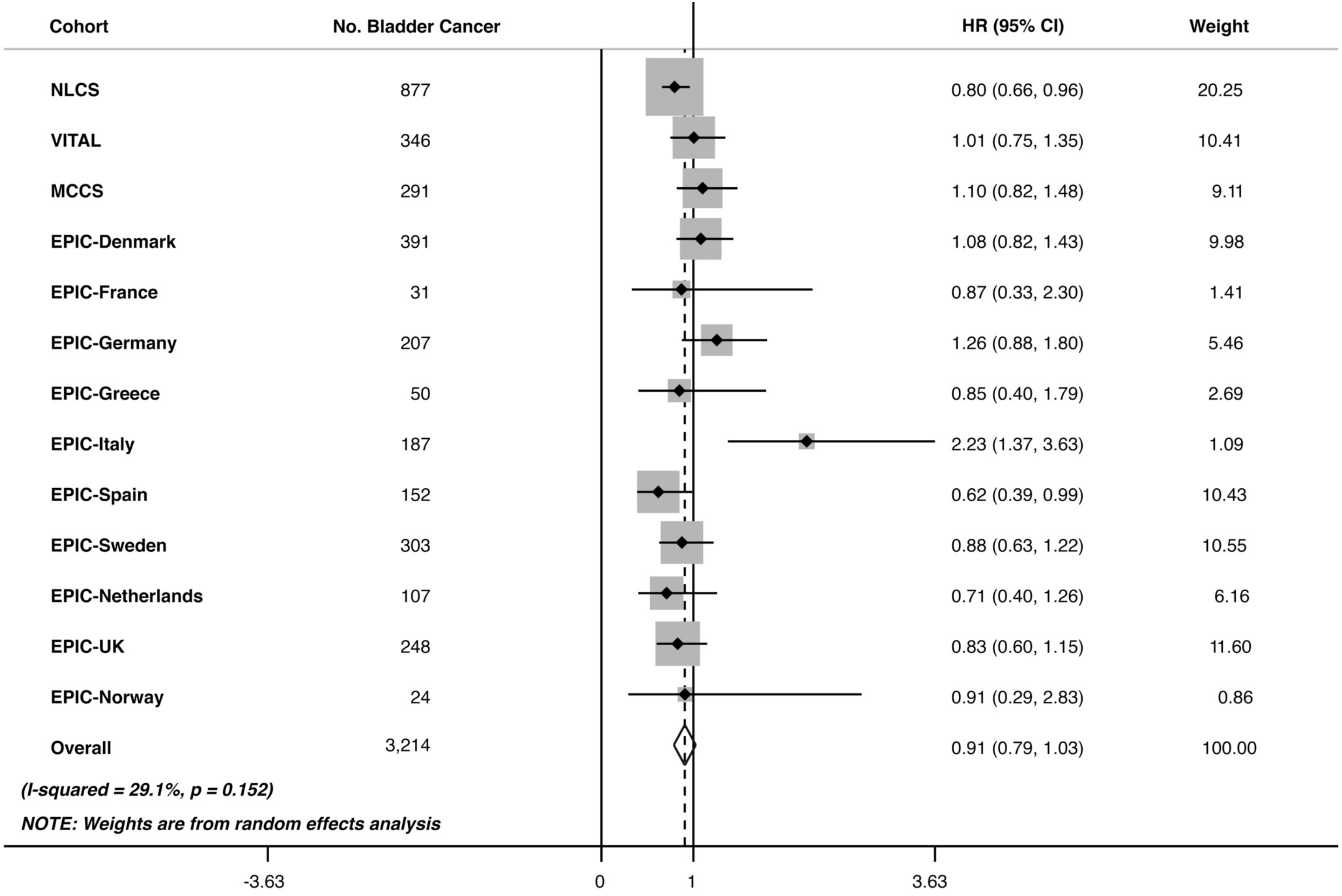

(I-squared = 29.1%, p = 0.152)

NOTE: Weights are from random effects analysis

Grains and dietary fiber intake and bladder cancer risk: a pooled analysis of prospective cohort studies
Yu et. al
On-line Supplementary Material

(C) Total Dietary Fiber

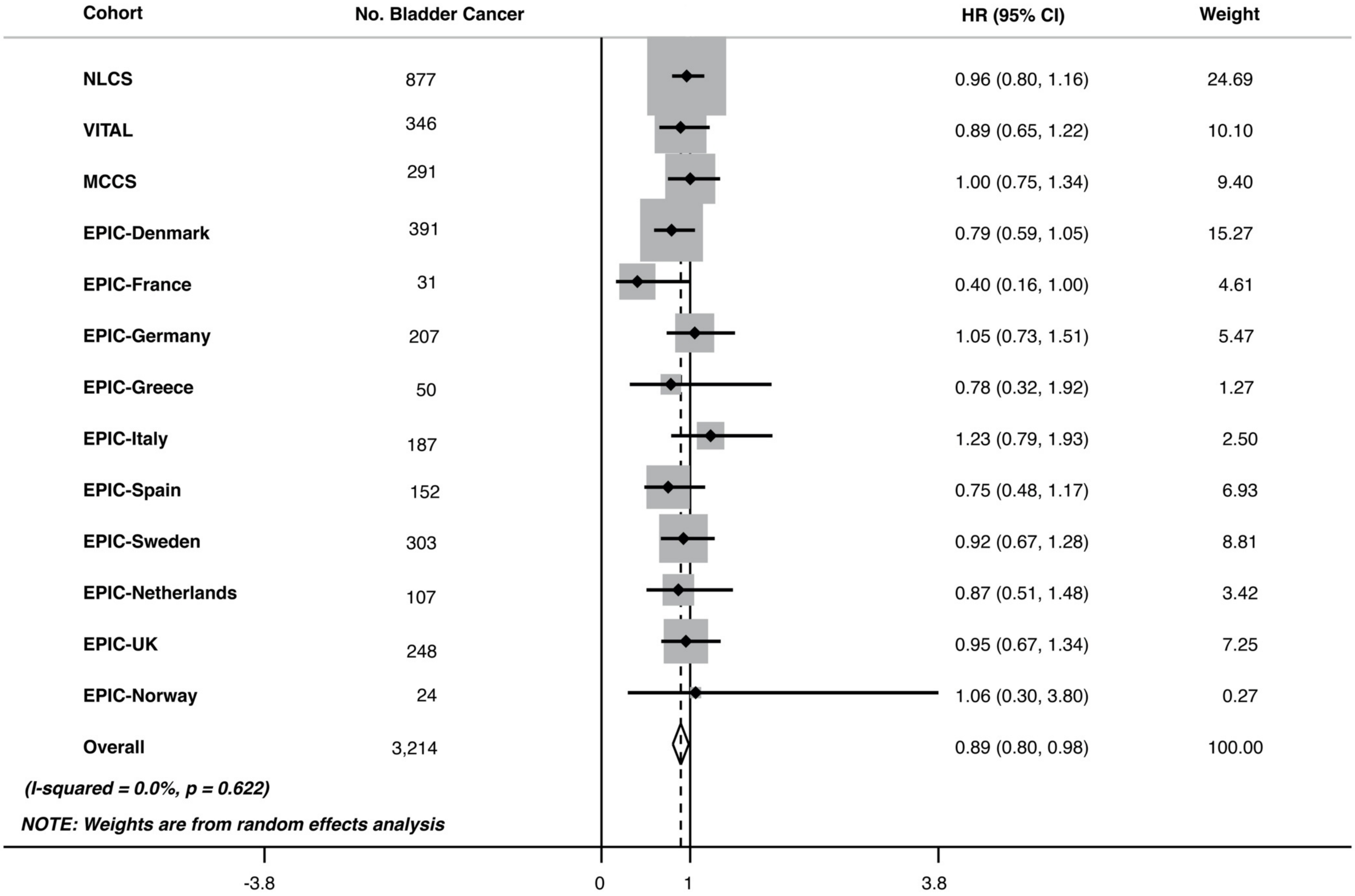

Grains and dietary fiber intake and bladder cancer risk: a pooled analysis of prospective cohort studies
Yu et. al
On-line Supplementary Material

(D) Cereal Fiber

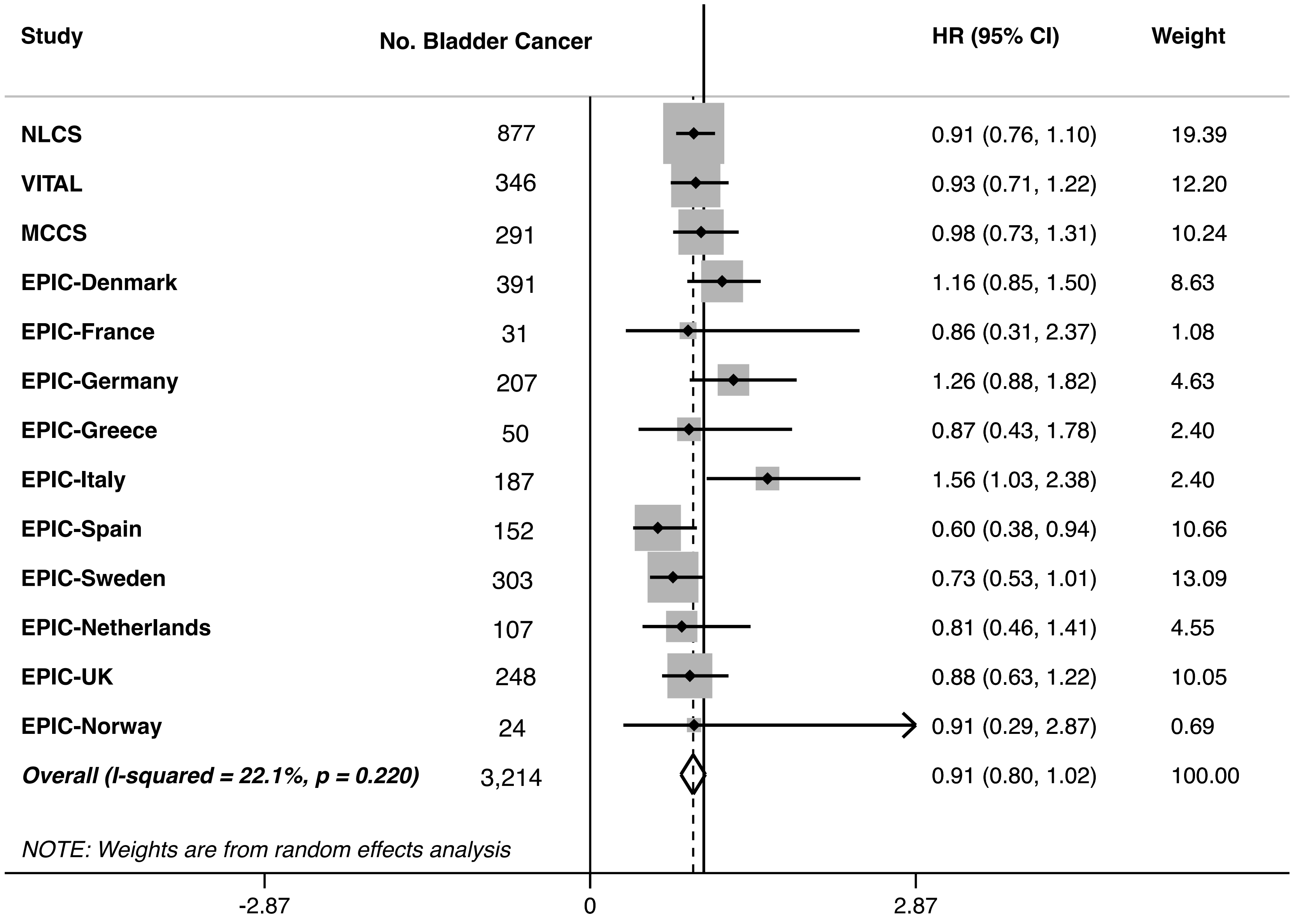

(E) Fruit Fiber

Study	No. Bladder Cancer	HR (95% CI)	Weight
NLCS	877	0.92 (0.78, 1.09)	32.40
VITAL	346	0.92 (0.70, 1.21)	11.72
MCCS	291	1.02 (0.76, 1.36)	8.33
EPIC-Denmark	391	0.98 (0.76, 1.28)	11.11
EPIC-France	31	0.78 (0.31, 2.00)	1.06
EPIC-Germany	207	1.02 (0.72, 1.43)	6.02
EPIC-Greece	50	1.97 (0.96, 4.05)	0.32
EPIC-Italy	187	0.99 (0.69, 1.41)	5.82
EPIC-Spain	152	1.28 (0.86, 1.92)	2.69
EPIC-Sweden	303	0.91 (0.67, 1.23)	9.58
EPIC-Netherlands	107	1.40 (0.86, 2.26)	1.55
EPIC-UK	248	0.89 (0.65, 1.23)	8.84
EPIC-Norway	24	0.98 (0.36, 2.67)	0.57
Overall (I-squared = 0.0%, p = 0.929)	3,214	0.96 (0.87, 1.05)	100.00

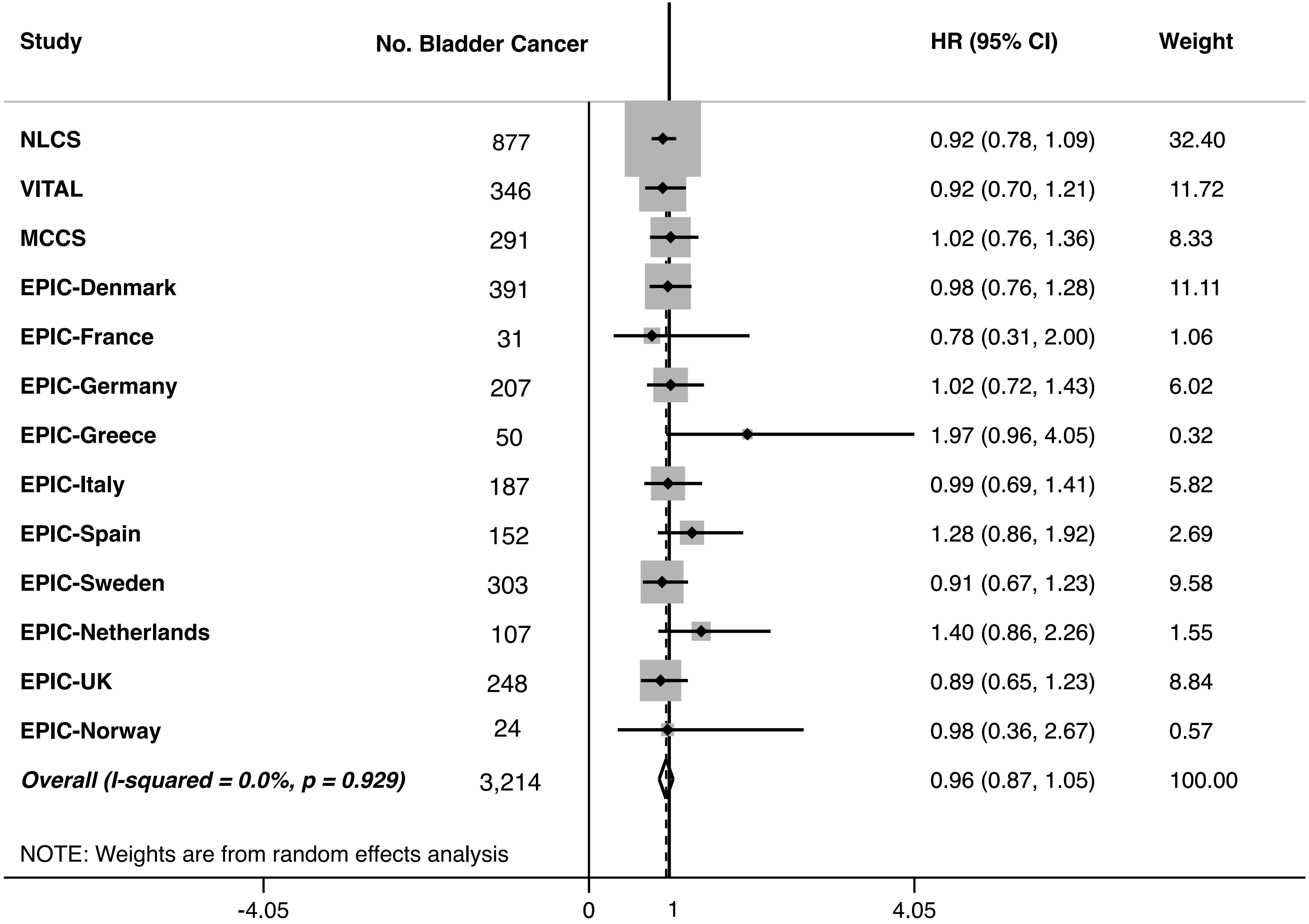

NOTE: Weights are from random effects analysis

Grains and dietary fiber intake and bladder cancer risk: a pooled analysis of prospective cohort studies
Yu et. al
On-line Supplementary Material

(F) Vegetable Fiber

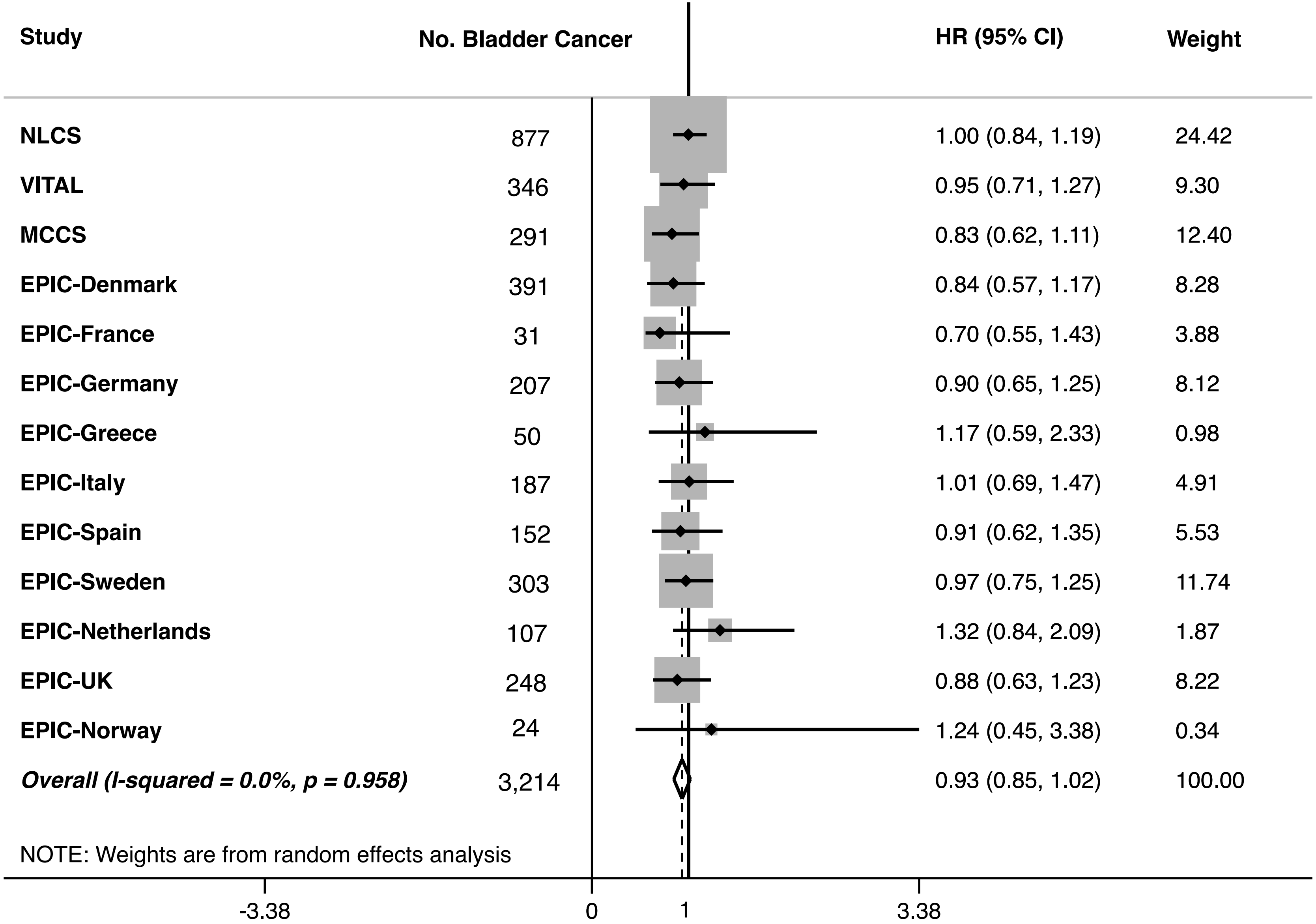

Supplementary Figure 3 Forest plot of meta-analyses with HRs and 95% CIs for highest vs. lowest intake of grain and dietary fiber with bladder cancer risk on (A) total whole grain; (B) total refined grain; (C) total dietary fiber; (D) cereal fiber; (E) fruit fiber; (F) vegetable fiber

Abbreviation: CI, confidence interval; g, gram; HR, hazard ratio; kcal, kilocalorie; ml, milliliter.

Diamond dots denote the hazard ratios (HRs); Horizontal lines represent the 95% confidence intervals (CIs); Weights (grey squares) are from random effects analyses.

Adjusted for age (years, continuous), sex (male or female), smoking (smoking was defined as: 0 (never smokers); 1 [current light smokers (*i.e.* smoking less than 20 pack-years)]; 2 [current heavy smokers (*i.e.* smoking more than 20 pack-years)]; 3 [current smokers (no information on pack-years)]; 4 [former light smokers (*i.e.* smokers who ceased smoking over 1 year prior and smoked less than 20 pack-years)]; 5 [former heavy smokers (*i.e.* smokers who ceased smoking over 1 year prior and smoked more than 20 pack-years)]; 6 [former smokers (smokers who ceased smoking over 1 year prior and no information on pack-years)]), total energy intake (kcal/day, continuous), ethnicity (Caucasian or non-Caucasian, if applicable) , alcohol intake (ml/day, continuous), fruit intake (g/day, continuous), fat intake (g/day, continuous), meat intake (g/day, continuous), sugar intake (g/day, continuous), vegetable intake (g/day, continuous), and total fluid intake (ml/day, continuous).

Reference group was lowest intake (tertile 1).

Supplementary Table 12 Risk of bladder cancer according to intakes of grain and dietary fiber after removing the study that most likely dominates the results (model 2)

Overall	Case/Total	Tertile 1	Tertile 2	Tertile 3	HR Per 1 SD Increase (95% CI)	*P*-trend	Removed Study
**Total Whole Drain (g/day)**	1,667/192,572	Ref.	1.00 (0.88, 1.14)	0.86 (0.76, 0.98)	0.95 (0.91, 0.99)	0.029	EPIC-Netherlands
**Total Refined Drain (g/day)**	3,027/529,522	Ref.	0.91 (0.83, 1.02)	0.89 (0.78, 1.01)	0.93 (0.88, 1.00)	0.051	EPIC-Italy
**Total Dietary Fiber (g/day)**	3,027/529,522	Ref.	0.91 (0.83, 1.00)	0.85 (0.74, 0.97)	0.90 (0.83, 0.97)	0.008	EPIC-Italy
**Cereal Fiber (g/day)**	3,027/529,522	Ref.	0.93 (0.84, 1.02)	0.90 (0.79, 1.01)	0.92 (0.87, 1.01)	0.067	EPIC-Italy
**Fruit Fiber (g/day)**	3,164/549,458	Ref.	0.97 (0.89, 1.07)	0.97 (0.86, 1.10)	0.98 (0.90, 1.06)	0.555	EPIC-Greece
**Vegetable Fiber (g/day)**	3,107/537,624	Ref.	0.99 (0.89, 1.09)	0.91 (0.79, 1.04)	0.90 (0.82, 0.99)	0.038	EPIC-Netherlands

Abbreviation: CI, confidence interval; g, gram; HR, hazard ratio; kcal, kilocalorie; ml, milliliter.

The intervals of tertiles were defined as; total whole grain: 0 ≤ tertile 1 ≤ 3 g/day, 3 < tertile 2 ≤ 8 g/day, tertile 3 > 8 g/day; total refined grain: 0 ≤ tertile 1 ≤ 102 g/day, 102 < tertile 2 ≤ 181 g/day, tertile 3 > 181 g/day; total dietary fiber: 0 ≤ tertile 1 ≤ 17 g/day, 17 < tertile 2 ≤ 25 g/day, tertile 3 > 25 g/day; cereal fiber: 0 ≤ tertile 1 ≤ 7 g/day, 7 < tertile 2 ≤ 12 g/day, tertile 3 > 12 g/day; fruit fiber: 0 ≤ tertile 1 ≤ 2 g/day, 2 < tertile 2 ≤ 4 g/day, tertile 3 > 4 g/day; vegetable fiber: 0 ≤ tertile 1 ≤ 5 g/day, 5 < tertile 2 ≤ 9 g/day, tertile 3 > 9 g/day.

[1, 2] Model 2 of Cox regression: Additionally, ethnicity (Caucasian or non-Caucasian, if applicable), alcohol intake (ml/day, continuous), fruit intake (g/day, continuous), fat intake (g/day, continuous), meat intake (g/day, continuous), sugar intake (g/day, continuous), vegetable intake (g/day, continuous) and total fluid intake (ml/day, continuous).

Reference group was lowest intake (tertile 1).

P-trend <0.05 was considered statistically significant.
